# Systemic acquired resistance: an emerging role for jasmonates in local signal biogenesis, translocation and distal signal decoding

**DOI:** 10.1111/nph.71405

**Published:** 2026-07-05

**Authors:** Fay Bennett, Erin Stroud, Pradeep Kachroo, Murray Grant

**Affiliations:** ^1^ School of Life Sciences University of Warwick Coventry CV4 7AL UK; ^2^ Department of Plant Pathology University of Kentucky Lexington KY 40546‐0312 USA

**Keywords:** effector‐triggered immunity, induced systemic resistance, jasmonates, long‐distance electrical signalling, systemic acquired resistance

## Abstract

Plant systemic acquired resistance (SAR) requires generation and movement of mobile signals from local leaves in which plant disease resistance has been activated (effector‐triggered immunity, ETI), to distal, uninfected regions, where they prime host defences. Although salicylic acid (SA) and N‐hydroxypipecolic acid (NHP) are widely recognised as key regulators of SAR, the requirement for *de novo* synthesis implies the existence of upstream or parallel inducing signals. Recent studies using whole plant and confocal reporter imaging, electrical signalling, and single‐cell transcriptomics have revealed how specific cell types and the temporal–spatial organisation of phytohormone networks contribute to immune signalling. We summarise these findings alongside previous knowledge to highlight the collective importance of jasmonates, calcium, reactive oxygen species, and electrical signals as early initiators, coordinators, and most likely propagators of long‐distance signalling during ETI‐induced SAR. We draw parallels with induced systemic resistance and highlight the coordinated roles of jasmonates, volatile compounds, and the microbiome in plant‐to‐plant communication. Furthermore, we also review environmental modulation of defence responses, a research area deriving further attention. Evidence points towards the coordinated activation of multiple signals, including jasmonates, driving systemic immunity across biological scales from the infected cell to entire plant communities.

## Graphical abstract

Jasmonate signalling underpins establishment of plant systemic acquired resistance (SAR). Activation of the hypersensitive response (HR) not only contains the pathogen but establishes broad spectrum resistance in unchallenged leaves. The strong burst of reactive oxygen/nitrogen species initiating the HR results in lipid peroxidation (illustrated by biophotons), providing substrate for jasmonate synthesis. The HR responding leaf develops spatially separated zones, those closest to the pathogen are enriched for salicylic acid (SA) and the distal for jasmonic acid (JA) biosynthetic genes. The jasmonate responsive reporter *JISS1::LUC*, captures rapid jasmonate translocation to distal tissues, elevating SA, critical for effective SAR.

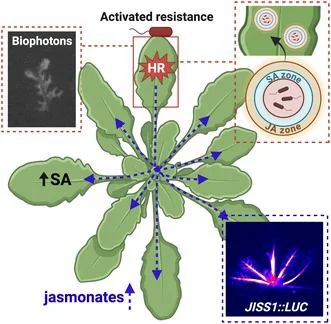

**Table:** 

	Contents	
	Summary	3142
I.	Introduction	3143
II.	Signalling in the local challenged leaf – generation of SAR‐eliciting signals	3145
III.	ETI‐generated chloroplast‐derived ROS drives lipid peroxidation and jasmonate synthesis in infected tissues	3146
IV.	Spatial regulation of SA‐JA antagonism during ETI explains the SA/JA antagonism conundrum	3147
V.	Jasmonates are critical for activation of systemic defence	3148
VI.	Single‐cell transcriptomics reveal different ‘resting states’ for core components of systemic signals	3148
VII.	Jasmonates, GLRs, electrical signals, and long‐distance translocation of systemic signals	3150
VIII.	Microbiome‐mediated and interplant SAR, an established role for jasmonates	3150
IX.	The impact of the environment on SAR–light regulation and circadian rhythms	3153
X.	Conclusions and future directions	3154
	Acknowledgements	3155
	References	3155

## I. Introduction

Plants lack an adaptive immune system with circulating mobile immune cells; thus, unlike mammalian cells, every plant cell must be capable of detecting and responding to microbial attack. To achieve this, a range of mechanisms are deployed, from pre‐formed defences such as chemical (phytoanticipins) and physical barriers through to induction of complex defence mechanisms post attack. Initial perception of danger is elaborated through a complex network of largely membrane/transmembrane‐associated receptors that perceive ‘non‐self’. These pattern recognition receptors (PRRs: see Table 1, for a complete list of abbreviations) not only perceive microbe‐ or pathogen‐associated molecular patterns (M/PAMPs) but also include damage (damaged‐self)‐associated molecular patterns (DAMPs) arising from, for example necrotrophic pathogen attack or herbivore attack (HAMPs). PRR responses are often highly conserved (Bjornson *et al*., 2021), triggering a basal immune response, commonly referred to as PAMP‐triggered immunity (PTI) or DTI/HTI depending on the specific challenge perceived, generally providing sufficient protection. However, host‐adapted pathogens can deliver a range of proteinaceous effectors or small molecules into plant cells and subvert innate immunity, resulting in effector‐triggered susceptibility (ETS) (Martel *et al*., 2021).

**Table 1:** Abbreviations.

(Microbe/pathogen/damage/herbivore)‐associated molecular pattern	(M/P/D/H)AMP
(PAMP/DAMP/HAMP) triggered immunity	(P/D/H)TI
Adenosine diphosphate‐ribose	ADPR
Arbuscular mycorrhizal fungi	AMF
Azelaic acid	AzA
Coiled coil	CC
Common mycelial network	CMN
CC‐NLR	CNL
(Chloroplastic) reactive oxygen species	cROS
Extracellular adenosine triphosphate	eATP
Extracellular nicotinamide adenine dinucleotide (phosphate)	eNAD(P)
Ethylene	ET
Effector triggered immunity	ETI
Effector triggered susceptibility	ETS
Photosystem II quantum efficiency	*F*_v_/*F*_m_
Glycerol‐3‐phosphate	G3P
Glutamate	Glu
Glutathione	GSH
Hydrogen peroxide	H_2_O_2_
Hypersensitive response	HR
Induced systemic resistance	ISR
Jasmonic acid	JA
Jasmonic acid‐isoleucine	JA‐Ile
Localised acquired resistance	LAR
Methyl salicylate	MeSA
(Microbial) volatile organic compound	mVOC
Nicotinamide adenine dinucleotide	NAD^+^
Nicotinamide adenine dinucleotide phosphate	NADPH
N‐hydroxypipecolic acid	NHP
Nucelotide‐binding domain leucine‐rich repeat	NLR
Nitric Oxide	NO
Superoxide	O_2_^−^
Plant growth‐promoting rhizobacteria	PGPR
Pipecolic acid	Pip
Primary immune responder	PRIMER
Pattern recognition receptor	PRR
Resistance	R
Helper NLR	RNL
Reactive oxygen species	ROS
Salicylic acid	SA
Systemic acquired resistance	SAR
Systemic‐induced surface potential	SISP
Slow wave potential	SWP
Trans‐acting small interfering RNA	tasiRNA
Toll/interleukin‐1 receptor	TIR
TIR‐CNL	TNL
Wound‐activated surface potential	WASP

**Fig. 1:**
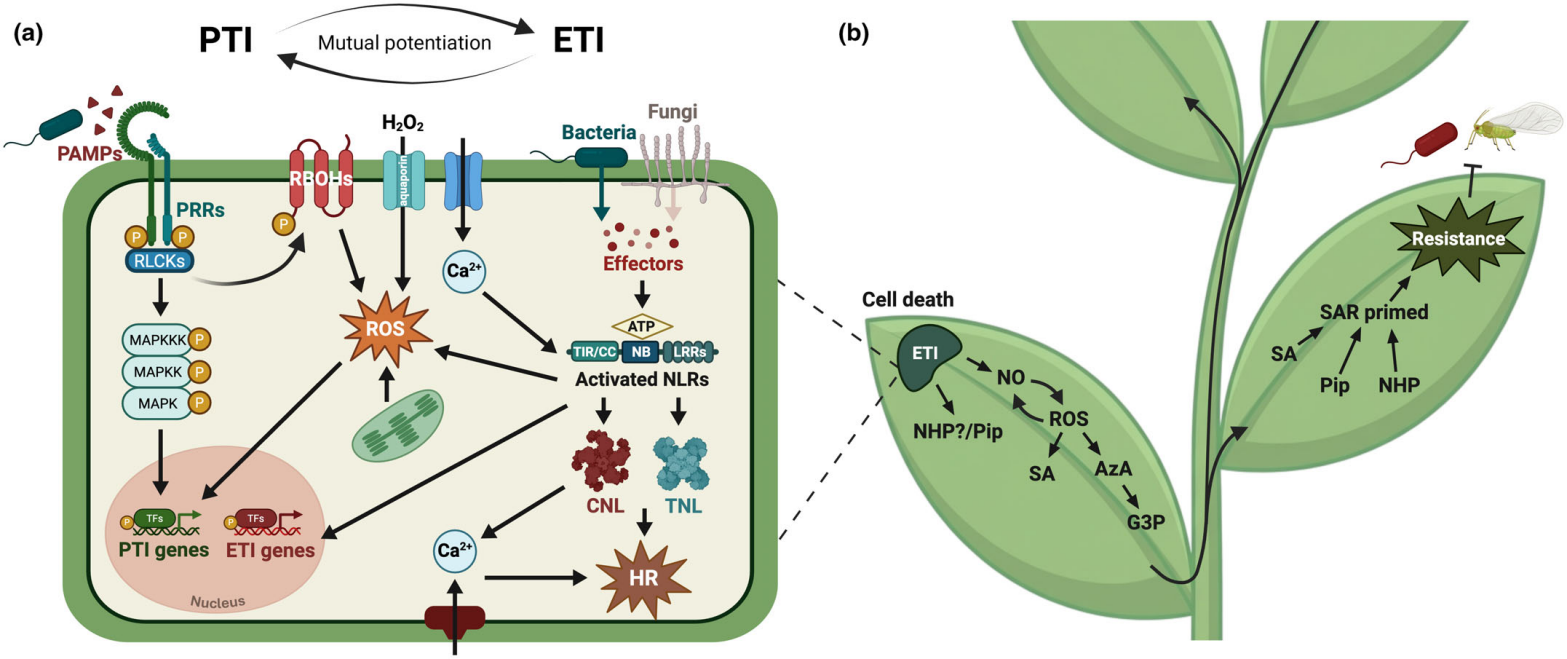
Schematic overview of key mechanisms involved in the development of systemic acquired resistance. (a) Perception and recognition of foreign infection molecules lead to activation of pattern‐triggered immunity (PTI). Injection of pathogen effectors activates effector‐triggered immunity (ETI), potentiates PTI, and triggers calcium influx and the induction of the hypersensitive response (HR). (b) Activation of a signal cascade in the infected leaf triggers ETI (dark green) via mechanisms described in (a), and production of key systemic acquired resistance (SAR) defence metabolites which upregulate defence signalling and prime distal systemic tissue against biotic challenge. AzA, azelaic acid; Ca^2+^, calcium; CNL, coiled‐coil nucleotide‐binding domain leucine‐rich repeat; G3P, glycerol‐3‐phosphate; H_2_O_2_, hydrogen peroxide; MAPK, MITOGEN‐ACTIVATED PROTEIN KINASE; NHP, N‐hydroxy‐pipecolic acid; NLR, Nucleotide‐binding leucine‐rich repeat; NO, nitric oxide; PAMPS, pathogen‐associated molecular patterns; Pip, pipecolic acid; PRR, pattern‐recognition receptors; RBOH, RESPIRATORY BURST OXIDASE HOMOLOG; RLCK, receptor‐like cytoplasmic kinases; ROS, reactive oxygen species; SA, salicylic acid; TNL, Toll/interleukin‐1 receptor (TIR) nucleotide‐binding domain leucine‐rich repeat. Figure created with BioRender Bennett, F. (2026) (BioRender.com/io5z98g).

To counter this, plants have a second layer of immunity conferred by plant disease resistance proteins – intracellular nucleotide‐binding domain leucine‐rich repeat (NLR) immune receptors. These directly or indirectly recognise effectors or their activities/cellular perturbations to activate an enhanced immune response. This ‘effector triggered immunity’ (ETI) is usually associated with a localised programmed cell death, the hypersensitive response (HR), that is thought to contain the pathogen (Fig. 1a). NLRs are further classified by their N‐terminal domain into coiled‐coil (CC)‐NLRs (CNLs) and Toll/interleukin‐1 receptor (TIR)‐NLRs (TNLs). Remarkable progress over the last 7 yr has revealed that perception of pathogen effectors or their activities leads to NLR oligomerisation by induced proximity of the N‐terminal CC or TIR domains, forming a resistosome that directly or indirectly facilitates immune signalling. Oligomerised TNLs form a tetrameric resistosome (Martin *et al*., 2020), the two asymmetric TIR homodimers forming a functional nicotinamide adenine dinucleotide (NAD^+^) hydrolase. TIR domains catalyse NAD^+^, DNA/RNA, and NTPs into a remarkably diverse range of small metabolites, including adenosine diphosphate‐ribose (ADPR), cyclic ADPR, and 3′ ribofuranosyladenosine, some of which activate CC domain ‘helper’ NLRs (RNLs, RPW8‐domain NLRs) via the core enhanced disease susceptibility (EDS1) immunity hub (Zhao *et al*., 2026). In Arabidopsis, the main helper RNLs are activated disease resistance 1 and N‐requirement gene 1 (NRG1s), which primarily support TNL sensors. CNLs can form remarkably diverse resistosomes, ranging from the HOPZ‐activated resistance 1(ZAR1) pentamer to the octamer formed by the CC_G10_‐NLR resistance to *Pseudomonas syringae* 2 (RPS2). By contrast, Solanaceae have specialised CNL‐type helpers (NRCs; required for cell death) that integrate signals from a massive network of many different ‘sensor’ NLRs, for which to date, hexameric and dodecameric resistosomes have been described. However, a unifying feature underpinning the HR is the formation of Ca^2+^ channels by CC domains of CNLs or RNL/NRC resistosomes, eliciting a sustained increase in cytosolic Ca^2+^, one of the earliest physiological events in the ETI‐mediated HR (see Kim *et al*., 2026 and Maruta *et al*., 2026, for comprehensive reviews). Emerging evidence indicates these resistosomes target different organelles, with the RNL, NRG1 shown to associate with single‐ and double‐membrane organelles via its N‐terminal RPW8‐like domain (Ibrahim *et al*., 2026).

Plant‐induced ETI can confer long‐lasting, broad‐spectrum resistance to bacteria, viruses, fungi, oomycetes, and herbivores, a process referred to as systemic acquired resistance (SAR) and more recently ‘trained immunity’ (Conrath, 2025). Nevertheless, our understanding of SAR signal initiation, propagation, and perception in systemic tissue remains fragmentary. While PTI‐induced SAR has been reported (Mishina & Zeier, 2007), so has systemic induced susceptibility (Cui *et al*., 2005). This review focusses predominantly on emerging signalling mechanisms underpinning ETI‐induced SAR, although we recognise that all SAR‐inducing challenges are established in the background of P/H/DTI responses; thus, mutual potentiation between PTI and ETI (Yan *et al*., 2025) likely acts to reinforce SAR.

The history of SAR research (Chester, 1933; Klessig *et al*., 2018) can be traced back to independent findings by Ray and Beauverie showing that prior pathogen infection results in an enhanced capacity to resist subsequent infections. Ross (1961) demonstrated that SAR was graft transmissible. The subsequent 60 yr revealed numerous additional SAR signalling components (Vlot *et al*., 2021; Spoel & Dong, 2024), comprising a variety of diverse compounds including azelaic acid (AzA) (Jung *et al*., 2009), glycerol‐3‐phosphate (G3P) (Chanda *et al*., 2011), dehydroabietinal (Chaturvedi *et al*., 2012), pipecolic acid (Pip) (Návarová *et al*., 2012), the actual bioactive component later identified as N‐hydroxy‐pipecolic acid (NHP) (Hartmann *et al*., 2018), nitric oxide (NO) (Wang *et al*., 2014), and monoterpenes (Riedlmeier *et al*., 2017) (Fig. 1b). More recently, *trans*‐acting small interfering RNAs (tasiRNAs) (Shine *et al*., 2022) and extracellular NAD (phosphate) (eNAD(P)) (Wang *et al*., 2019; Li *et al*., 2023) have emerged as novel SAR regulators.

The diverse plant–pathogen models used in SAR research make understanding and comparing the timing of SAR initiation and signal translocation challenging. For example, infiltration of the apoplastic bacterial pathogen, for example *Pseudomonas syringae* pv *tomato* strain DC3000, enables relatively synchronous activation of infection (albeit bypassing ‘stomatal immunity’) and allows multiple specific resistance proteins to be studied. By contrast, fungal or oomycete challenges typically have slower and more complex asynchronous infection strategies combined with larger effector complements. Yet, even when deploying the same pathosystem, comparability between laboratories is impacted by a range of variables including inoculum concentration, time of immunising challenge, temperature, humidity, daylength, and light quality.

### 1. SA and NHP‐ the ‘key’ players in SAR

SA and NHP are the most intensively studied SAR signals, acting interdependently via a mutual amplification loop. Pathogen‐induced local SA accumulation can trigger NHP synthesis, and both are regulated by the transcription factors SAR Deficient 1 and Calmodulin‐Binding Protein 60 g (CBP60g). NHP‐induced transcriptional reprogramming and establishment of SAR requires the SA receptor Nonexpressor of Pathogenesis‐Related Genes 1 (NPR1) and its interacting bZIP (basic region leucine zipper) family TGA (TGACG‐binding) transcription factors (Yildiz *et al*., 2021). Both NHP and SA are conjugated into inactive forms by the same glycosyltransferase, UGT76B1 (Bauer *et al*., 2021; Mohnike *et al*., 2021). While the accumulation of SA in distal tissues is essential for SAR, this elevation depends on regulated transport rather than simple diffusion. As a weak acid (pKa 2.9), SA exists primarily in its protonated, membrane‐permeable form within the mildly acidic apoplast, becoming deprotonated and effectively sequestered upon entry into the more alkaline cytosol. Recent evidence suggests that SA is actively loaded into the apoplast, potentially via proton‐pump‐mediated gradients, and translocated distally through a process influenced by transpiration and cuticular integrity as well as cellular pH (Lim *et al*., 2020; Xia *et al*., 2026). By contrast, NHP is highly mobile. Both SA and NHP are synthesised *de novo*, thus requiring an upstream inducing signal. To date, knowledge of the mechanistic processes associated with SAR signal generation in local ETI responding leaves, signal translocation (or bifurcation), and decoding in the systemic responding tissues are rudimentary, and at best fragmentary.

While SAR provides broad‐spectrum resistance to pathogens with diverse lifestyles, few studies rigorously test this attribute. Usually, a virulent pathogen of the same strain used for immunisation is tested to evaluate the effectiveness of SAR. This limited experimental testing of diverse pathogens fails to adequately assess SAR's key biological outcome – broad‐spectrum immunity to biotrophic, hemibiotrophic, or necrotrophic pathogens, which colonise disparate host tissues, from roots to leaves, and occupy distinct apoplastic, epidermal or vascular ‘habitats’. Given such complexity, it would be remarkable if a single signalling molecule, translocated from a local site of biogenesis, could reach systemic tissues in sufficient quantity for decoding and activate a defence response effective against such a vast array of virulence strategies.

**Fig. 2:**
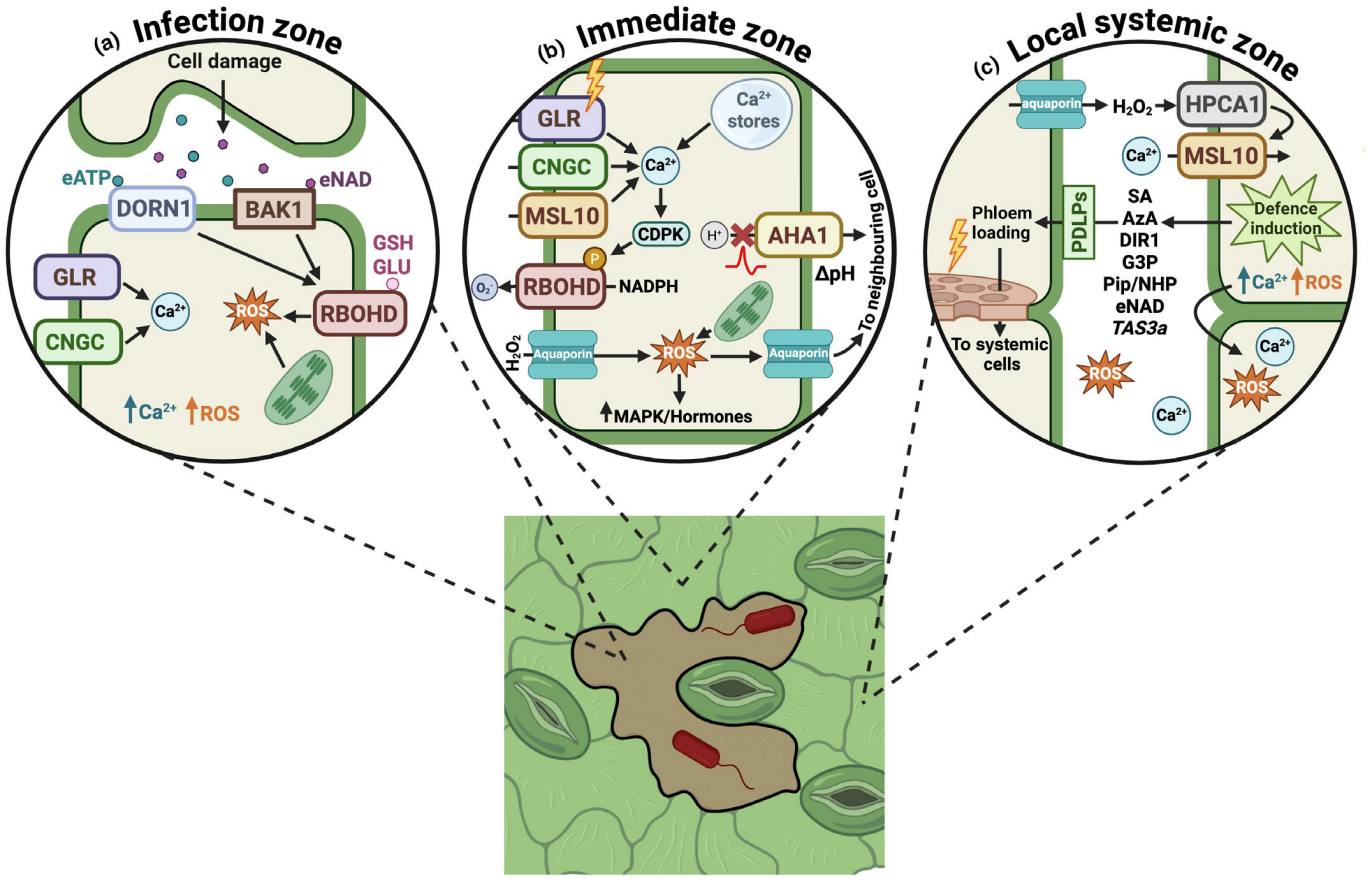
Induction of defence in the infected and nearby cells. Simplified schematic overview of the (a) mechanisms relating to damage signal (blue circle, eATP; purple circle; pink circle, GSH/Glu) perception in the infection zone, (b) defence signal generation in the ‘immediate zone of infection’, and (c) generation and translocation of systemic acquired resistance metabolites from cell‐to‐cell in the local systemic zone. AHA1, *Arabidopsis* H^+^‐ATPase 1; AzA, azelaic acid; BAK1, BRI1‐associated receptor kinase 1; Ca^2+^, calcium; CDPK, calcium‐dependent protein kinase; CNGC, Cyclic Nucleotide‐Gated Channel; DIR1, DEFECTIVE IN INDUCED RESISTANCE 1; DORN1, Does Not Respond to Nucleotides 1; eATP, extracellular adenosine triphosphate; eNAD, extracellular nicotinamide adenine dinucleotide; G3P, glycerol‐3‐phosphate; GLR, Glutamate Receptor‐Like channels; GLU, glutamate; GSH, glutathione; H_2_O_2_, hydrogen peroxide; HPCA1, HYDROGEN‐PEROXIDE‐INDUCED Ca^2+^ INCREASES 1; HR, hypersensitive response; MAPK, mitogen‐activated protein kinase; MSL10, mechanosensitive channel MscS‐LIKE 10; NADPH, nicotinamide adenine dinucleotide phosphate (reduced); NHP, N‐hydroxy‐pipecolic acid; PDLP, plasmodesmata‐localized proteins; RBOHD, RESPIRATORY BURST OXIDASE HOMOLOGs; ROS, reactive oxygen species; SA, salicylic acid; O_2_^−^, superoxide. Figure created with BioRender, Bennett F. (2026) (BioRender.com/cgyq8vi).

This review focusses on recent discoveries in ETI‐elicited SAR signalling; however, we reiterate this is within the context of wounding, microbial, or herbivore challenge of the plant cell, which inevitability causes cellular damage, albeit at different scales. While colonisation by biotrophic pathogens causes limited cellular damage at the site of entry, necrotrophic pathogens actively kill plant tissue. By contrast, insects employ diverse feeding strategies, ranging from minimal impact stylet‐based sucking and piercing to macroscopic rasping and chewing behaviour, generating diverse HAMPs (Snoeck *et al*., 2022). Regardless of the mechanism, and independent of ETI, pathogen colonisation initially triggers recognition of PAMPs; the induction of HAMP/DAMP‐triggered immunity or active secretion of phytokines, such as systemin (Schilmiller & Howe, 2005), or even apoplastic pH changes (Wang *et al*., 2026) can elicit an underlying immune response (extensively reviewed by Hou *et al*., 2019; Snoeck *et al*., 2022; Shu *et al*., 2023). Activation of these PRRs is often underpinned by specific Ca^2+^ signatures, not necessarily solely derived from the apoplast via active transport but also from intracellular stores including the vacuole, endoplasmic reticulum, chloroplasts, and mitochondria (Köster *et al*., 2022). Fig. 2 illustrates some key early infection events before, or concomitant with, ETI elicitation and SAR activation in the immunised leaf. We highlight several DAMPs linked to both mechanical and microbial induced damage with documented roles in systemic immunity.

## II. Signalling in the local challenged leaf – generation of SAR‐eliciting signals

### 1. A core role for jasmonates as activators of SAR signalling?

Initially ETI induces accumulation of SAR elicitors (including Pip, NHP, AzA, tasiRNAs, and G3P) in infected and, to a lesser extent, distal tissues leading to the activation of systemic defence (extensively reviewed by Vlot *et al*., 2021; Spoel & Dong, 2024). Given that most SAR‐associated signals are synthesised *de novo*, the initial transcriptional mechanisms that induce these elicitors across the plant remain poorly understood. Here, we highlight an emerging body of evidence that positions jasmonates not merely as SA antagonists but as integral components in the elicitation of SAR signals.

The model for reciprocal antagonism between SA and jasmonic acid (JA) in plant defence, reviewed by Thaler *et al*. (2012), underpins a long‐standing evidence‐based framework positing SA signalling being activated at the expense of the JA signalling (Spoel & Dong, 2008; Aerts *et al*., 2021; Hou & Tsuda, 2022). However, exceptions exist, notably lack of strong antagonism between these pathways in systemic tissue following challenge with different biotic stressors, hinting that perhaps spatial regulation could impact hormonal crosstalk (Spoel *et al*., 2007). Several recent reports implicating jasmonates as both initiators and propagators of ETI‐induced SAR necessitate re‐evaluating SA‐JA antagonism in SAR (Betsuyaku *et al*., 2018; Gaikwad *et al*., 2026; Li *et al*., 2026). We first mine key historical biochemical and physiological evidence then integrate recent spatial gene expression and imaging evidence, both of which circumvent destructive sampling, to provide temporal–spatial information and a previously unexpected role for *de novo* jasmonate synthesis leading to initiation of SAR signalling. A notable caveat is these data are primarily derived from studies on bacterial elicited ETI, predominately predicated on the rapid activation of the CNL, RPM1, which induces intracellular Ca^2+^ increases within 2 h post infection (hpi) (Grant *et al*., 2000). This model system allows study of synchronous activation of rapid ETI signalling, either in partial or whole leaf challenges with a characterised SAR or hormonal reporters.

## III. ETI‐generated chloroplast‐derived ROS drives lipid peroxidation and jasmonate synthesis in infected tissues

**Fig. 3:**
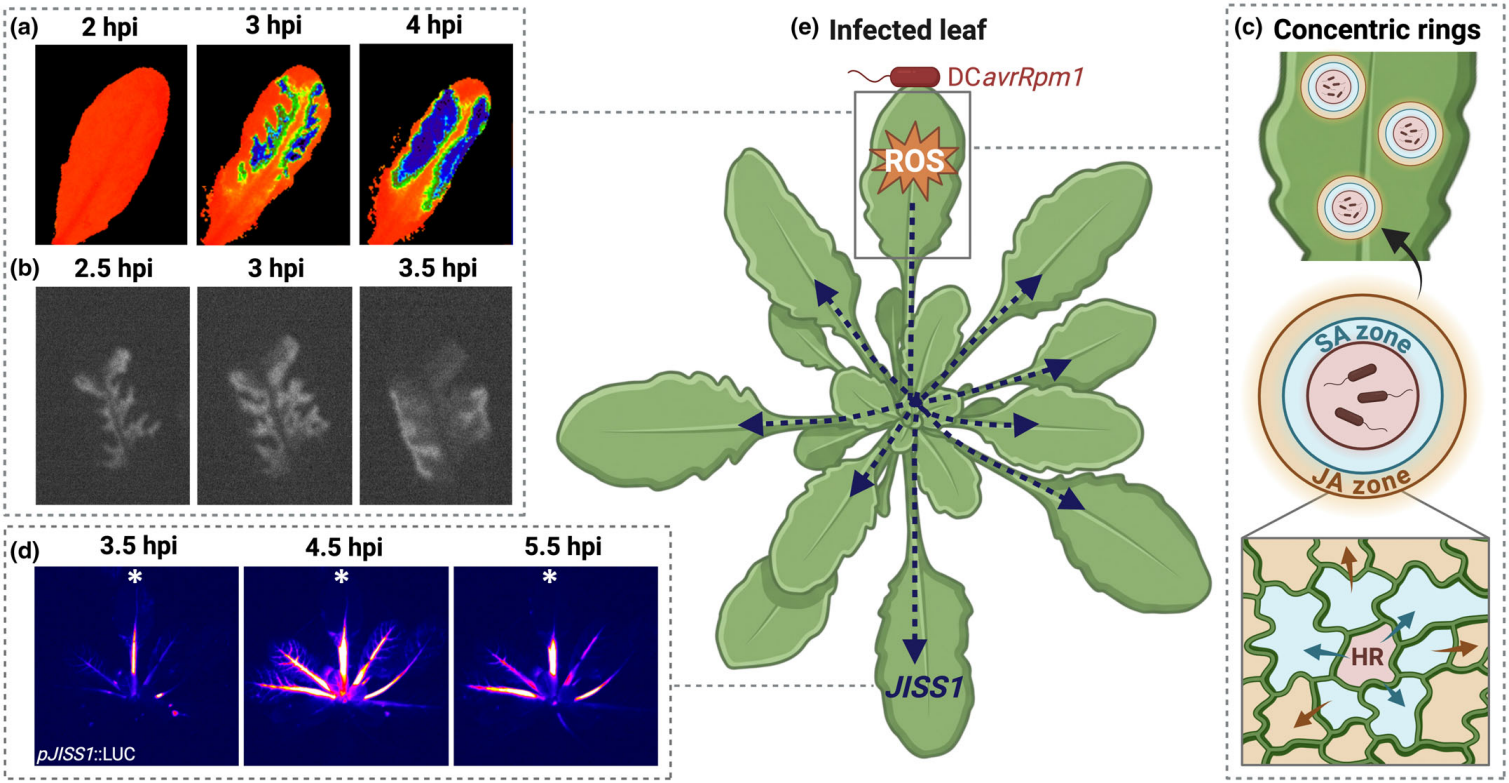
Key signalling events occurring following infection with effector‐triggered immunity (ETI)‐inducing avirulent pathogen DC*avrRpm1* in Arabidopsis. (a) Photosystem II quantum efficiency (*F*_v_/*F*_m_) imaging time course captures early *Arabidopsis thaliana* chloroplast changes in response to *Pseudomonas syringae* pv *tomato* strain DC3000 (DC) carrying *avrRpm1* (DC*avrRpm1*) from 2 to 4 h post infection (hpi). Healthy tissue is red, tissue ultimately showing hypersensitive response (HR) cell death is blue. (b) Lipid peroxidation captured as a wave of biophoton emission in the infected *A. thaliana* leaf between 2.5 and 3.5 hpi. (c) Concentric rings of salicylic acid (SA) (blue) and jasmonic acid (JA) (orange) form around the infection site (HR) following activation of effector triggered immunity. (d) *JASMONATE‐INDUCED SYSTEMIC SIGNAL 1*::LUCIFERASE reporter (*pJISS1::LUC:* see Gaikwad *et al*., 2026) signalling from a DC*avrRpm1* infected leaf (indicated with asterisk) at 3.5 hpi to systemic leaves throughout *A. thaliana* vasculature and epidermal cells at 4.5–5.5 hpi. (e) Panels (a–c) indicate ETI‐induced systemic acquired resistance signalling within an infected leaf. Panels (d, e) show *pJISS1::LUC* signalling to systemic tissues throughout the Arabidopsis plant. Figure created with BioRender, Bennett F. (2026) (BioRender.com/agr2fsn).

To understand how different ‘zones’ are initiated in the infected tissue, one needs to consider the role chloroplast‐derived ROS (cROS), primarily Photosystem II‐derived singlet oxygen (^1^O_2_), plays in ETI (Zurbriggen *et al*., 2010; Dmitrieva *et al*., 2020; Kachroo *et al*., 2021; Roussin‐Léveillée *et al*., 2025). The timing and extent of ETI‐elicited cROS can be captured by monitoring Chl fluorescence, with suppression of *F*_v_/*F*_m_ (maximum photochemical quantum yield efficiency of Photosystem II), providing surrogate temporal spatial dynamics (Littlejohn *et al*., 2021) (Fig. 3a). ^1^O_2_ is a major driver of lipid peroxidation, rapidly interacting with the chloroplast membrane, oxidising galactolipids, generating lipid peroxides, and reactive electrophile species that contribute to cell death signalling (Triantaphylidès *et al*., 2008). Critically, lipid peroxidation precedes the HR.

Keppler & Novacky (1987) reported lipid peroxidation during HR development in cucumber cotyledons challenged with incompatible *P. syringae* pv *pisi* 40 yr ago. Twenty years later, detailed biochemical analyses of *Arabidopsis* leaves challenged with incompatible DC3000*avrRpm1* demonstrated induction of 9‐ and 13‐lipoxygenase (*LOX2*)‐dependent oxylipin synthesis. The major oxylipins JA, 12‐oxo‐phytodienoic acid, dinor‐oxo‐phytodienoic acid, but not SA, rapidly accumulated within 2–4 hpi (Andersson *et al*., 2006), supporting an earlier study showing JA accumulation in developing tobacco HR lesions 3–8 hpi following incompatible *P. syringae* pv *phaseolicola* challenge (Kenton *et al*., 1999). A subsequent systematic lipidomics study reinforced the importance of lipid peroxidation during DC3000*avrRpm1*‐induced HR (Zoeller *et al*., 2012). Whereas ^1^O_2_ was the major contributor to lipid peroxidation under basal conditions, pathogen infection resulted in substantial increases in *LOX2* and free radical‐catalysed lipid oxidation. Interestingly, *lox2* mutants revealed a role for LOX2 in enzymatic membrane peroxidation but not for the pathogen‐induced free jasmonate production during the HR. Notably, this study also identified the SAR signalling molecule, AzA, as a general lipid peroxidation marker. Lipid peroxidation in leaves undergoing an incompatible interaction generates ultraweak photon emissions, which can be visualised as biophotons (Fig. 3b), providing temporal spatial information on lipid peroxidation dynamics during the HR (Bennett *et al*., 2005; Birtic *et al*., 2011).

## IV. Spatial regulation of SA‐JA antagonism during ETI explains the SA/JA antagonism conundrum

If jasmonates are important in SAR, how does one reconcile classical SA/JA antagonism? Initial clues come from a pioneering study on ETI in *Arabidopsis thaliana* expressing fluorescent reporters driven by the promoters of *PATHOGENESIS RELATED 1* (*PR1*) for SA or *VEGETABLE STORAGE PROTEIN 2* (*VSP2*) for JA (Betsuyaku *et al*., 2018). DC3000*avrRps2*‐elicited ETI led to ‘concentric rings’ of SA and JA expressing cells beyond the pathogen challenged tissue (Fig. 3c). An ‘SA ring’ consisting of a relatively small circle of cells bordering the developing HR lesion, recently coined the localised acquired resistance zone (Jacob *et al*., 2023). Immediately adjacent to the SA ring and extending across much of the outer leaf surface was a ring of JA‐expressing cells. While induction of both markers overlapped temporally (induced *c*. 9 hpi), spatial distinction was maintained across the time course. Recently, comprehensive spatial imaging using SA and JA biosynthetic or responsive marker genes (Li *et al*., 2026) elegantly documented spatial separation of SA and JA responses during DC3000*avrRpm1*‐activated ETI. Live cell imaging was undertaken on both low inoculum spray challenged 2‐wk‐old seedlings and high inoculum syringe infiltrated 4‐wk‐old Arabidopsis expressing fluorescent constructs reporting classical PTI (*pFRK1::NLS‐3xmVenus*), SA/JA biosynthetic gene expression (*CBP60g* and *AOS* respectively), or SA/JA‐responsive gene expression (*PR1* or *VSP1* respectively). RPM1 activated ETI elicited by low inoculum potentiated immunity in *A. thaliana* expressing the classical PTI reporter construct *pFRK1::NLS‐3xmVenus*, revealing spatially confined PTI in discrete ‘bundles’ surrounding viable DC3000 microcolonies. The dual fluorescent reporters revealed predominantly mutually exclusive activation across neighbouring cells forming adjacent inner (SA) and outer (JA) domains that become more distinct over time (Betsuyaku *et al*., 2018). Downstream hormone responses captured by *PR1* and *VSP1* reporter expression recapitulated similar spatial compartmentalisation around infected cells, with *PR1* expression enriched proximal to infection, whereas *VSP1* predominated in surrounding regions. This live cell imaging of multiple ‘concentric rings’ evolving in cells surrounding each bacterial colony complements and expands Betsuyaku *et al*.'s (2018) investigation of RPS2‐activated ETI at infection boundaries and aligns with recent spatial transcriptomics (Nobori *et al*., 2025). Both studies provide compelling evidence for spatially defined mutual induction of SA and JA pathways during ETI while accommodating the well‐documented requirement for local SA‐based defences, adding context to the somewhat counter‐intuitive jasmonate dependency for SAR signal generation, recently reported by Gaikwad *et al*. (2026). Indeed, spatial regulation of SA‐JA antagonism has also been demonstrated in various plants and cell types, including Arabidopsis root cells in response to challenge with the fungal pathogen *Fusarium oxysporum* (Calabria *et al*., 2025), and herbivory and wounding in potato (Levak *et al*., 2025). Despite the differences in these pathosystems, a consistent feature was the establishment of a central SA zone at the infection or wound site surrounded by an outer JA zone.

## V. Jasmonates are critical for activation of systemic defence

A role for jasmonates in classic ETI‐elicited SAR was proposed nearly 20 yr ago by Truman *et al*. (2007), following identification of an early responding systemic marker gene (*A70*, recently renamed *JISS1 – JASMONATE‐INDUCED SYSTEMIC SIGNAL 1*). JA signalling and biosynthetic genes were induced systemically within 4 hpi with DC3000*avrRpm1* and SAR was abolished in the JA biosynthetic *12‐oxophytodienoic acid reductase 3* (*opr3*) and JA signalling regulator *jasmonate‐insensitive 1* (*jin1/myc2*) mutants. Using a *JISS1::LUCIFERASE* reporter (hereafter *JISS1::LUC*) to dissect classical SA and SAR elicitor pathways, Gaikwad *et al*. (2026) highlighted an important role for jasmonates in SAR. *JISS1::LUC* activation was observed in response to RPM1‐elicited ETI in the infected leaf within 3.5 hpi, with the signal translocating down the petiole and into systemic tissue within 4.5 hpi (Fig. 3d). Local *JISS1::LUC* induction was observed in response to infiltration with JA, JA‐isoleucine, and its *P. syringae*‐derived bioactive mimic, coronatine, but not SA or other classic SAR elicitors. *JISS1::LUC* induction was totally abolished in the JA biosynthetic mutant *allene oxide synthase* (*aos*) and the JA receptor *coronatine insensitive 1* (*coi1*). Critically, and consistent with Truman *et al*. (2007), both these mutants lost the ability to activate SAR to virulent DC3000. Furthermore, RPM1‐induced *JISS1::LUC* signal induction was unaffected in several SA pathway mutants (*sid2*, *npr1*, and *nac15/nac55/nac72*), the NHP synthesis mutant *flavin‐containing monooxygenase 1* (*fmo1*), or following co‐infiltration with jasmonate pathway inhibitors. *JISS1::LUC* activity was induced by all tested incompatible bacterial challenges (both CNL and TNL), although temporal dynamics remained unique to the specific *Avr‐R* combination. Collectively, and supported by Chl fluorescence, lipid peroxidation, quantitative hormone profiling combined with local spatial transcriptomic and imaging (Fig. 3), it is evident jasmonates play a central role in initiation and establishment of SAR, preceding the induction of classic SAR markers and SA accumulation distally. These data firmly place jasmonates within the ETI‐induced SAR framework and *JISS1* as an early systemic defence marker (Polwatte Koralalage *et al*., 2026).

## VI. Single‐cell transcriptomics reveal different ‘resting states’ for core components of systemic signals

Complementing real‐time imaging, recent single‐cell spatial transcriptomics further support a critical role for different cell and induced cell states in plant immunity. Nobori *et al*. (2025) showed the importance of cell‐type heterogeneity using single‐cell transcriptomics, epigenetics, and spatial transcriptomics following activation of RPS2 in Arabidopsis. Analogous to the concentric rings of SA and JA expressing cells discussed previously (Li *et al*., 2026), two specialised cell states close to the infection site, ‘Primary Immune Responder (PRIMER)’ cells and very closely associated ‘bystander’ cells were identified (Nobori *et al*., 2025). PRIMER cells are rapidly activated by infection, in turn inducing downstream immune signalling pathways, influencing neighbouring bystander cells, which likely make spatially discrete contributions to the initiation of long‐distance immune signalling and hormone pathways.

**Fig. 4:**
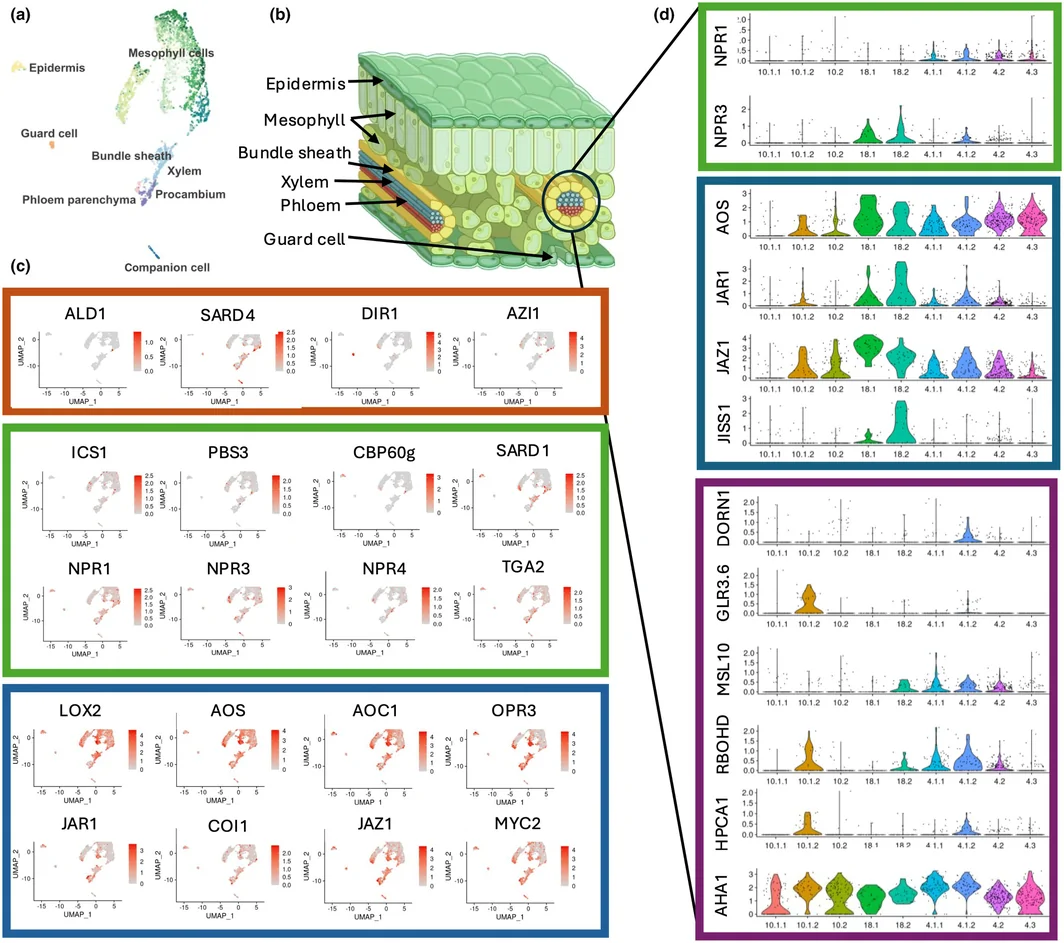
Single‐cell transcriptomics reveal distinct basal cellular localisation of systemic acquired resistance (SAR)‐related defence response genes. Expression of selected genes involved in the biosynthesis or regulation of the N‐hydroxypipecolic acid (NHP: red box), salicylic acid (SA: green box), or jasmonic acid (JA: blue box) pathways in unchallenged *Arabidopsis thaliana* leaf tissue, mined from the Plant Single Cell Browser online database (Ma *et al*., 2020; Kim *et al*., 2021). (a) Uniform Manifold Approximation and Projection (UMAP) of leaf cells displaying the 19 distinct clusters identified by Kim *et al*. (2021). (b) Diagram of leaf structure highlighting key cell types. (c) Expression of N‐hydroxypipecolic acid biosynthetic genes (red box: *ALD1* and SARD4) and lipid transfer proteins (red box: *DIR1* and *AZI1*), SA (green box: *ICS1, PBS3, CBP60g, SARD1, NPR1, NPR3, NPR4*, and *TGA2*), and JA (blue box: *LOX2, AOS, AOC1, OPR3, JAR1, COI1, JAZ1*, and *MYC2*) genes in leaf tissue. Expression levels are indicated by red and the localisation of cellular expression maps to the UMAP in (a). (d) Expression of SA (green box: *NPR1* and *NPR3*), JA (blue box: *AOS, JAR1, JAZ1, JISS1*), and other defence associated genes (*DORN1, GLR3.6, MSL10, RBOHD, HPCA1*, and *AHA1*) in enriched vasculature tissue. Violin plots display normalised gene expression of the cells (demonstrated as dots) within vasculature cell types corresponding to the C4 (Bundle Sheath, Xylem), C18 (Phloem parenchyma, Xylem), and C10 (Phloem parenchyma, Procambium) subclusters identified in (a). Data sourced from https://www.zmbp‐resources.uni‐tuebingen.de/timmermans/PSCB3_leaf/. Panel (b) adapted from BioRender, Bennett F. (2026) (BioRender.com/n1heqgx).

Emerging single cell datasets enable interrogation of the basal transcriptional activity of specific genes, whose signal in specific cell types may be markedly diluted by destructive sampling. To help reconcile discrete JA/SA contributions to immunity, and specifically SAR, we mined the online Plant Single Cell Browser (Ma *et al*., 2020; Kim *et al*., 2021) to establish the ‘native’, uninduced cell‐type expression of components involved in SAR signal generation such as SA, NHP, and JA (Fig. 4). Interestingly, essential genes involved in the biosynthesis and regulation of SA and NHP, and the lipid transfer proteins *DIR1 and AZI1* involved in the translocation of G3P and AzA, show very little expression in the uninduced state. Notable expression of SA receptors *NPR1* and *NPR3* was only observed in the vasculature, but within distinct cell types, likely reflecting their opposing roles in regulating the SA signalling (Ding *et al*., 2018), but also potentially JA signalling. The importance of vasculature is reflected by specific subclusters of ETI responding phloem companion cells being enriched with immune‐related genes, for example subcluster 8 specifically expressing the NHP biosynthetic genes *ALD1* *(AGD2‐LIKE DEFENSE RESPONSE PROTEIN 1)* and *FMO1* (Nobori *et al*., 2025; Nobori, 2025). By contrast, basal levels of JA biosynthetic and regulatory genes displayed significant expression across multiple cell types including the vasculature. A similar broad patterning was observed in other defence‐associated genes including the proton pump *AHA1* (Kumari *et al*., 2019), ROS‐producing *RBOHD*, and the H_2_O_2_ receptor *HPCA1* (Wu *et al*., 2020). Others, including the eATP receptor *DORN1 (DOES NOT RESPOND TO NUCLEOTIDES 1)* (Kundu *et al*., 2025) and as expanded on below, *GLUTAMATE RECEPTOR‐LIKE (GLR) GLR3*.3 and *GLR3.6* involved in long‐distance signalling in response to wounding and herbivory (Mousavi *et al*., 2013; Toyota *et al*., 2018), display more specialised localisation. Collectively, these data support a distinction in the ‘resting’ states of the SA, NHP, and JA signalling pathways, with jasmonate and some other defence mechanisms (including ROS and Ca^2+^ pathways) displaying more broadly activated states within the uninduced cell (Stroud *et al*., 2022).

## VII. Jasmonates, GLRs, electrical signals, and long‐distance translocation of systemic signals

The first evidence for the potential contribution of jasmonates to long‐distance biotic communication is derived from a pivotal publication by Mousavi *et al*. (2013) demonstrating that localised leaf wounding generates electrical signals termed Wound‐Activated Surface Potentials (WASPs), which travel systemically to distal leaves, leading to the induction of the JA pathway repressor *JASMONATE‐ZIM DOMAIN 10* (*JAZ10*) and accumulation of the bioactive jasmonate JA‐isoleucine. WASPs are variation potentials, also referred to as slow wave potentials (SWPs) (Barbosa‐Caro & Wudick, 2024), dependent on the nonselective cation channels *GLR GLR3.3* and *GLR3.6* (Mousavi *et al*., 2013; Nguyen *et al*., 2018). The mechanisms underlying how electrical signals trigger systemic jasmonate accumulation is an active area of ongoing research. Models include Ricca's factors (Gao *et al*., 2023) and the squeeze‐cell hypothesis posed by Farmer *et al*. (2014). The latter postulates that wounding induces rapid changes in xylem pressure, which spread radially, ‘squeezing’ surrounding xylem‐contact cells, inducing jasmonate biosynthesis genes, as exemplified by *LOX6* expression, via clade 3 *GLR's*. Ricca's factors were recently identified as glutamate and/or short‐lived aglycone intermediates, derived from aliphatic glucosinolates via xylem insect feeding or wounding‐induced mobile β‐THIOGLUCOSIDE GLUCOHYDROLASEs 1 & 2 (Gao *et al*., 2023). These activated Ca^2+^ signalling and antiherbivory defences in distal tissues. Critically, loss of these signals abolished distal *JAZ10* induction highlighting a potential role in systemic JA accumulation.

Since the discovery of WASPs, similar GLR3.3‐ and GLR3.6‐dependent variation potentials have been reported in plant long‐distance communication (Grenzi *et al*., 2021) and environmental stress (GLR3.4: Meyerhoff *et al*., 2005). GLRs also play an important role in plant immunity, including activation of PTI (GLRs2.7/2.9 Bjornson *et al*., 2021), NLR activation (Wang *et al*., 2025), systemic immunity against fungal (Manzoor *et al*., 2013), bacterial pathogens (Wang *et al*., 2025; Gaikwad *et al*., 2026), herbivory (Toyota *et al*., 2018), and insect egg recognition (GLR2.7; Mineiro *et al*., 2025). How these GLRs are activated is a key area of interest (Suda & Toyota, 2022), and recent studies have highlighted wound‐activated release of glutamate and other amino acids which, acting as DAMPs, are bound by *GLR3.3* and *GLR3.6* to initiate systemic Ca^2+^ signalling (Toyota *et al*., 2018; Bellandi *et al*., 2022). Even locally, Ca^2+^ channel formation by coil‐coil domains of CNLs and/or RNLs/NRCs are potentially bolstered by GLR2.9 (Wang *et al*., 2025). These multifaceted roles of Ca^2+^ in plant immunity, both locally and systemically, have been recently reviewed (Gilroy, 2025; Sun *et al*., 2026).

### 1. Electrical signalling in SAR

**Fig. 5:**
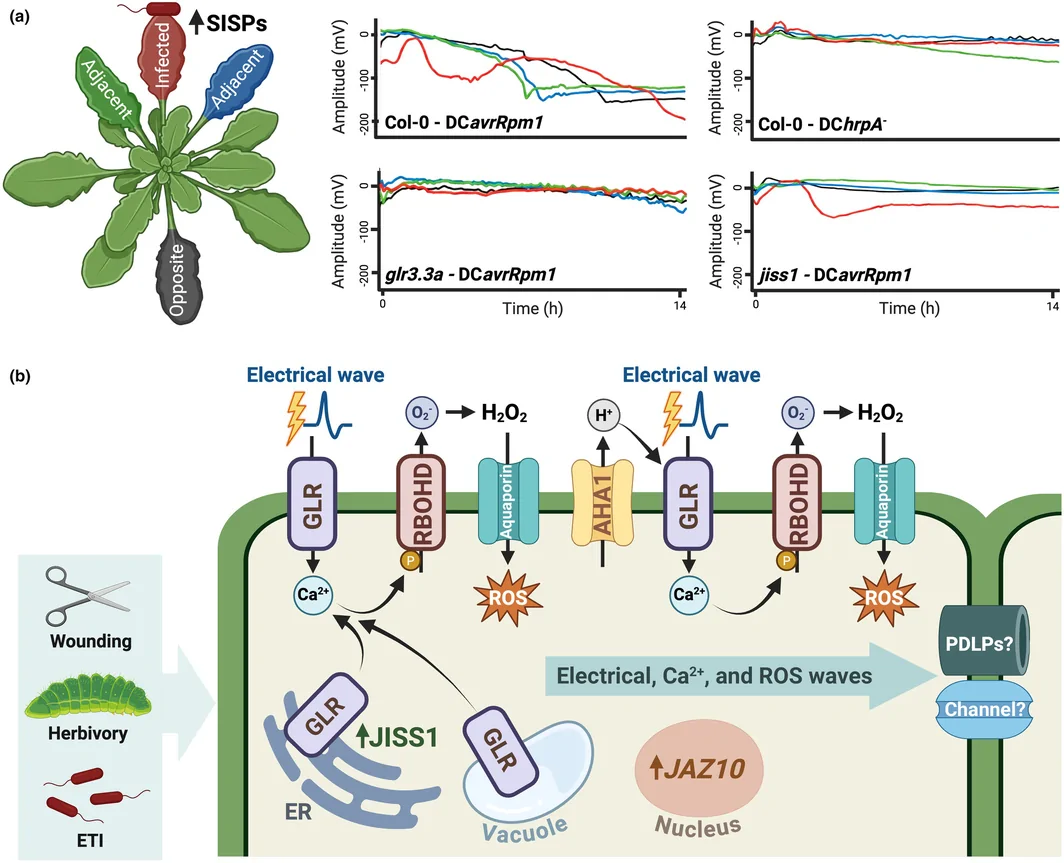
Schematic overview of key electrical and chemical waves that propagate systemically cell‐to‐cell. (a) *Pseudomonas syringae* pv *tomato* strain DC3000 (DC) carrying *avrRpm1* (DC*avrRpm1*) challenged *Arabidopsis thaliana* leaf (red) shows initial depolarisation at *c*. 2 h post infection (hpi) (red trace), generating systemic induced surface potentials (SISPs) in adjacent leaves (blue leaf and blue trace and green leaf and green trace) *c*. 4–7 hpi, and later in the opposite distal leaf (black leaf and black trace). Depolarisation displayed as changes in amplitude (mV) over time (hours). No SISPs are generated in the DC*hrpA* ^*−*^ mutant (T3SS‐deficient strain). The *glutamate receptor 3.3a* mutant (*glr3.3a*) does not propagate SISPs in response to DC*avrRpm1*, nor does the *jasmonate induced systemic signal 1* mutant (*jiss1*). Graphs illustrate representative data trends derived from Gaikwad *et al*. (2026). (b) Wounding, herbivory, and ETI‐induced cell death activate a complex signalling network underpinned by electrical, reactive oxygen species (ROS), and calcium waves propagated cell‐to‐cell. AHA1, *Arabidopsis* H^+^‐ATPase 1; ETI, effector triggered immunity; ER, endoplasmic reticulum; H_2_O_2_, hydrogen peroxide; GLR, Glutamate Receptor‐Like channels; JAZ10, JASMONATE ZIM‐DOMAIN 10; JISS1, JASMONATE‐INDUCED SYSTEMIC SIGNAL 1; PDLP, Plasmodesmata‐Located Proteins; RBOHD, RESPIRATORY BURST OXIDASE HOMOLOGs; O_2_^−^, superoxide. Figure created with BioRender, Bennett F. (2026) (BioRender.com/f86rvrb).

Gaikwad *et al*. (2026) noted remarkable similarity in spatial patterning between the *JISS1::LUC* reporter and wound responses (Mousavi *et al*., 2013), both having a common underpinning jasmonate signalling requirement. Indeed, RPM1 induced ETI activated variation potentials in systemic leaves (termed systemic‐induced surface potentials, SISPs), albeit manifested at a much slower rate than WASPs (Fig. 5a); however, they required an underlying PTI component as conditional induction of *AvrRpm1* alone failed to recapitulate DC3000*avrRpm*1‐induced SISPs. Notably, ETI failed to induce SISPs in the *jiss1* mutant, and this phenotype was recapitulated in the *coi1* JA receptor mutant and both *glr3.3* and *glr3.6* mutants. Critically, *JISS1::LUC* responded to local wounding, and systemic induction of *JISS1* was lost following co‐infiltration of DC3000*avrRpm1* with Ca^2+^ inhibitors (Truman *et al*., 2007). These data provide a mounting body of evidence implicating jasmonates, Ca^2+^, electrical signals, and GLRs in the induction of rapid, long‐distance signals during plant defence (Fig. 5b).

Intriguingly, existing single‐cell transcriptomics data predict colocalisation of *JISS1* and *GLR3.3* to the endoplasmic reticulum of phloem sieve elements and *GLR3.6* predominantly expressed in xylem contact cell tonoplast membranes (Ma *et al*., 2020; Kim *et al*., 2021; Morin *et al*., 2023; Gaikwad *et al*., 2026). Notably, JASMONATE TRANSPORTER 3 and 4 (JAT3 and JAT4), whose transcripts are expressed predominantly in companion cells and phloem parenchyma, interact with GLR3.3 to regulate phloem loading of JA during the wound response (Li *et al*., 2020). These colocalisation inferences suggest that SAR responses engage similar signalling pathways to wounding, being dependent upon jasmonates and electrical signalling, underpinned by GLRs. While JISS1 is absolutely required for SISPs, how SISPs are decoded and their specific contribution to SAR remains to be addressed. Although *JISS1::LUC* is a remarkably early, spatial–temporal marker for JA‐dependent systemic defence induction, *jiss1* displayed normal SAR to bacterial challenge (Gaikwad *et al*., 2026). Potential functional redundancy between *JISS1* homologues remains to be assessed. One may speculate that these very early immune responses, encompassing JA, Ca^2+^ signalling, ROS, lipid peroxidation and electrical signals represent bifurcations in a SAR signalling network which encodes inbuilt redundancy, wherein one pathway is unlikely to underpin full establishment of defence. This model allows SA, NHP, AzA, G3P, and other established SAR elicitors to operate in the same network as JA and other early signals, leading to a broad‐spectrum, robust response.

## VIII. Microbiome‐mediated and interplant SAR, an established role for jasmonates

While the role of jasmonates in foliar ETI‐induced SAR is less defined, the role for jasmonates in belowground rhizosphere host defence activation and interplant SAR at an environmental scale is arguably more firmly established.

### 1. Belowground rhizosphere defence communication

**Fig. 6:**
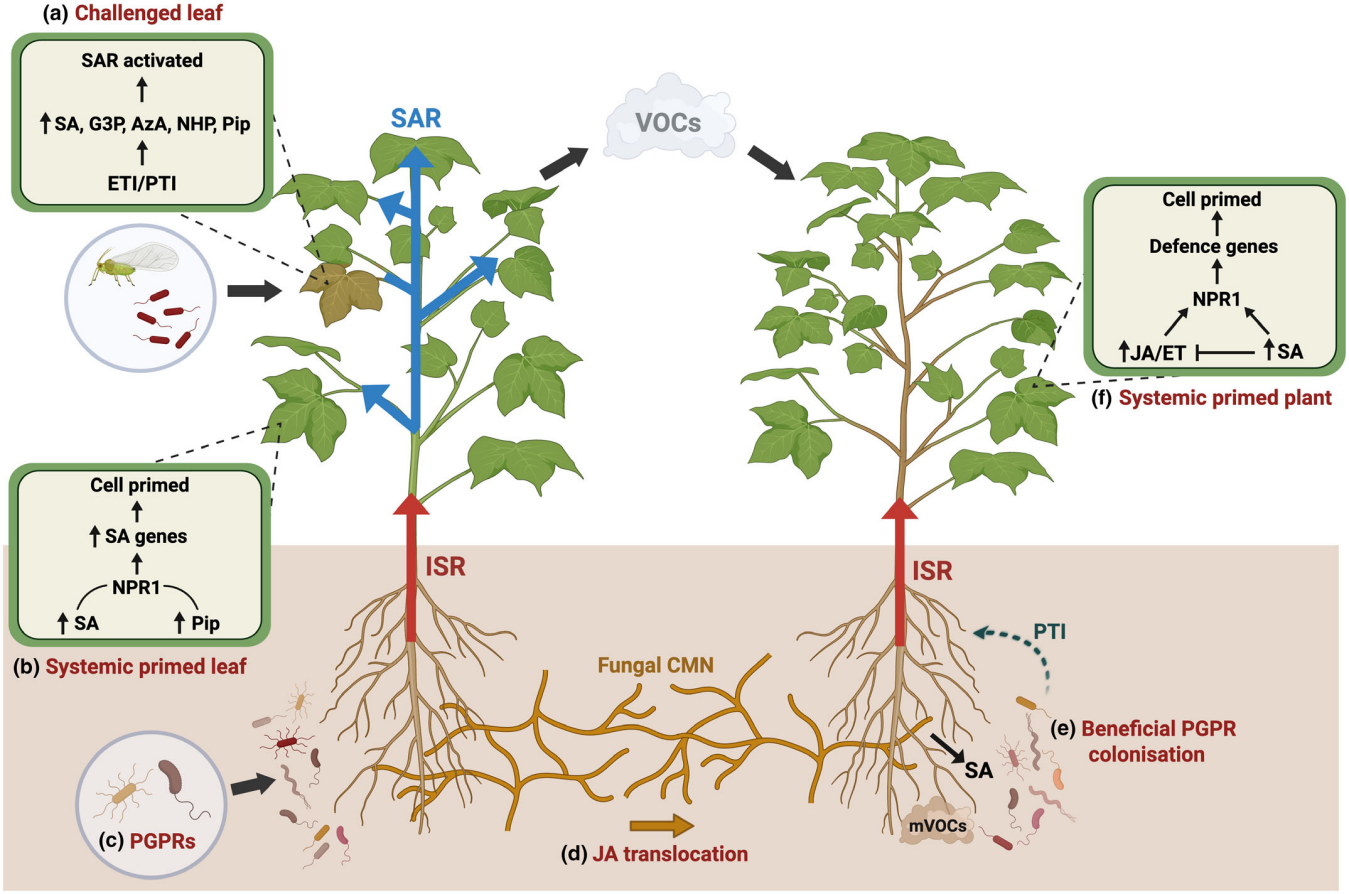
Schematic of systemic resistance at the environmental scale. (a) Infection leads to activation of pattern‐triggered/effector‐triggered immunity (PTI/ETI), upregulating key systemic acquired resistance (SAR) metabolites. (b) Upregulation of defence in systemic tissue primes against potential attack. Volatile organic compounds (VOCs) are released into the atmosphere and prime neighbouring plants. (c) Colonisation of beneficial rhizosphere microorganisms activates low‐level PTI and induced systemic resistance (ISR). (d) Jasmonic acid (JA) and stress metabolites move through fungal common mycelial networks between plants, inducing JA in neighbours. (e) Release of salicylic acid (SA) into the soil encourages similar beneficial rhizophore microbiota between plants, also activating ISR. (f) A combination of SAR and ISR induced responses leads to upregulation of defence hormones and priming of neighbouring species for potential attack. AzA, azelaic acid; CMN, common mycelial network; ET, ethylene; G3P, glycerol‐3‐phosphate; mVOCs, microbial volatile organic compounds; NHP, N‐hydroxy‐pipecolic acid; NPR1, NON‐EXPRESSOR OF PR GENES 1; PGPR, plant growth‐promoting rhizobacteria; Pip, pipecolic acid; PTI, pattern‐triggered immunity; . Figure created with BioRender, Bennett F. (2026) (BioRender.com/g5ryl0u).

The rhizosphere, a diverse nutrient‐rich ecosystem within the soil surrounding plant roots, is home to a large microbial community that maintains a dialogue with its ‘host’ plant. Beneficial rhizosphere microorganisms, such as the plant growth‐promoting rhizobacteria (PGPR) *Bacillus* spp., *Streptomyces* spp., and *Pseudomonas* spp., or fungi such as *Trichoderma* spp., can induce a primed immune state to confer broad‐spectrum disease resistance (Peer *et al*., 1991; Yu *et al*., 2022), also known as ‘induced systemic resistance’ (ISR). While SAR is commonly induced by foliar pathogenic microbes following ETI activation (Fig. 6a,b), ISR primes resistance in plants induced by beneficial nonpathogenic microorganisms in the soil (Fig. 6c). Root‐localised PRRs recognise foreign MAMPs, such as flagellin and lipopolysaccharides, activating formation of coreceptor complexes to phosphorylate downstream substrates. This leads to increased cytosolic Ca^2+^, ROS generation, and MAPK cascade activation analogous to PTI activation (Pršić & Ongena, 2020). However, PTI induced by PGPR is weaker, enabling establishment of a mutually beneficial relationship with the plant host (Su *et al*., 2024). Examples of common PGPR inducing ISR against pathogens have been extensively reviewed (Pršić & Ongena, 2020; Rabari *et al*., 2023). Unlike SAR, where metabolites such as SA, NHP, and methyl salicylate have been discovered as key mobile signals, no single universal mobile signal has been identified to confer ISR. ISR was first reported independently by three research groups in 1991, demonstrating induced resistance by PGPR to halo blight in common bean (Alström, 1991), *Colletotrichum orbiculare* in cucumber (Wei, 1991), and Fusarium wilt in Carnation, *Dianthus caryophyllus* L. (Peer *et al*., 1991). The subsequent 30 yr has revealed that secretion of beneficial enzymes (Zhang *et al*., 2025), volatile organic compounds (VOCs) (Wenig *et al*., 2019; Brosset & Blande, 2022), and metabolites (Kong *et al*., 2021) into the rhizosphere by a variety of PGPR's helps boost plant immunity, enhancing biotic stress tolerance to phytopathogens and pests (reviewed in Yu *et al*., 2022; Zhou *et al*., 2021).

In contrast to conventional models of SAR, ISR is primarily associated with rhizosphere‐driven SA‐independent, JA/ethylene (JA/ET)‐based defence priming (Pieterse *et al*., 1996; van Loon *et al*., 1998). However, multiple reports of dual activation of SA and JA/ET pathways by PGPR indicate that these complex overlapping signalling mechanisms often converge (Fig. 6f) (Ilham *et al*., 2019; Yuan *et al*., 2019; Samaras *et al*., 2021). Notably, both SAR and ISR are blocked in the *npr1* mutant in Arabidopsis (Cao *et al*., 1994; Pieterse *et al*., 1998), a key regulator of downstream defence genes in both SA and JA pathways. Simultaneous activation of both SAR and ISR results in an additive effect against virulent *Pst*DC3000 in an NPR1‐dependent manner (van Wees *et al*., 2000), indicating synergistic overlap between both pathways. Activation of transcription factor gene MYB72 in Arabidopsis roots when colonised with *Pseudomonas fluorescens* (WCS417r) bacteria induced a JA/ET‐dependent ISR (Van der Ent *et al*., 2008). Intriguingly, a recent study also found a root‐to‐shoot signalling defence mechanism, by which the glycosyltransferase UGT76B1 modulates *FMO1* synthesis of NHP through immobilisation, to control the level released into the shoot and enhance defence activation (Xu *et al*., 2025). This regulatory circuit is altered by *Trichoderma* endophytic and *Fusarium* biotrophic fungi, through modulation of UGT76B1 and *FMO1*, highlighting a common actively regulated feedback pathway linking both ISR and SAR (Xu *et al*., 2025).

### 2. Communication through common mycorrhizal networks (CMNs)

Intriguingly, SAR can also be established via plant‐to‐plant communication through belowground fungal common mycelial networks (CMNs). Endomycorrhizal arbuscular mycorrhizal fungi (AMF) of the *Glomeromycota* phylum are the most widespread amongst plant taxa, colonising *c*. 80% of terrestrial plant species (Brundrett & Tedersoo, 2018). AMF hyphae penetrate the plant host root epidermis and colonise cortical cells through the formation of arbuscules. These facilitate the bidirectional transport of carbon from the host plant and essential inorganic nutrients from the fungi, in a symbiotic mutualism (Wipf *et al*., 2019). Through anastomosis, AMF fuse together to form a CMN, connecting plants of the same or multiple species and notably increasing plant tolerance to abiotic stress including drought (Forczek *et al*., 2022) and salt stress (Boorboori & Lackóová, 2025), herbivory (Babikova *et al*., 2013; Song *et al*., 2014), and pathogens (Zhang *et al*., 2025). This underground communication serves as a highway for allelochemical information, warning neighbouring ‘receiver’ plants of imminent attack and allowing for primed defences to increase resistance.

Early studies found increased disease resistance in tomato plants connected by CMN infected with leaf early blight *Alternaria solani* (Song *et al*., 2010), indicating potential for transfer of disease resistance via CMN. Shortly thereafter, an increased release of herbivore‐induced plant volatiles in undamaged neighbouring *Vicia faba* plants was reported (Babikova *et al*., 2013), indicating systemic defensive priming also occurred through CMN. JA‐responsive genes upregulated in receiver plants connected to methyl jasmonate‐treated donor plants demonstrated JA signalling is required for CMN‐defence mediated communication (Song *et al*., 2014). More recently, JA was confirmed to be actively moving through CMN from *Botrytis cinerea*‐infected tomato to healthy receivers to help prime their defence system (Fig. 6d), while indirectly promoting root colonisation of disease‐suppressive rhizobacteria (Zhang *et al*., 2025) (Fig. 6e). Interplant communication of defensive metabolites mediated via CMNs extends the concept of SAR beyond the individual plant to a primed immune network occurring at the community level.

### 3. Plant volatile organic signals

Plants can defensively prime both its own distal leaves outside of the orthostichy (Frost *et al*., 2007) and neighbouring plants through release of VOCs into the atmosphere (Song & Ryu, 2018). Many plant VOCs have been documented in response to both biotic (Song & Ryu, 2018) and abiotic stresses (Cofer *et al*., 2018), with the aerial chemical composition varying depending on the initiating stimuli. The expansive variety of VOCs currently identified has been extensively reviewed by Brosset & Blande (2022).

Analysis of VOCs released following activation of the CNL RPM1 in the *eds1* Arabidopsis mutant background revealed an essential role for the monoterpenes, α‐pinene, β‐pinene, and camphene as signalling intermediates (Riedlmeier *et al*., 2017). Exposure to pinenes triggered ^1^O_2_ generation and upregulated AzA *INDUCED*1 (*AZI1*) and *EARLY ARABIDOPSIS ALUMINIUM INDUCED1* in an SA‐dependent manner in the infected plants, while also enhancing resistance to *P. syringae* pv *tomato* (*Pst*) in neighbouring plants. These monoterpene VOCs signal via Pip, G3P, and *LEGUME LECTIN‐LIKE PROTEIN1 (LLP1*), functioning in a positive feedback loop to activate SAR both between neighbouring plants and within plants (Wenig *et al*., 2019). Similarly, the plant volatile nonanal, an aldehyde that can be oxidised into the C9 fatty acid nonanoic acid, was found to reduce virulence of *Pst* in lima bean plants neighbouring those with chemically induced SAR (Yi *et al*., 2009), while exposure of *A*. *thaliana* to isoprene and ß‐caryophyllene respectively induced SA‐ and JA‐dependent defences against DC3000 (Frank *et al*., 2021). In tomato, β‐caryophyllene released by inoculation of *Bacillus amyloliquefaciens* altered the rhizophore microbiota of neighbouring receiver plants through increased release of SA from roots (Kong *et al*., 2021) (Fig. 6e). These diverse examples highlight an important yet complex role for plant VOCs in promoting a beneficial rhizosphere microbiome across ecosystems. Superimposed on this, microbes also produce microbial VOCs (mVOCs) to influence plant growth and systemic resistance (Ali *et al*., 2025). The potential for detection of plant health status using VOC emission detection methods has already been demonstrated (Patel *et al*., 2023), with biotic stress detection undergoing field trials (Fuentes *et al*., 2021).

## IX. The impact of the environment on SAR–light regulation and circadian rhythms

The trade‐off between abiotic and biotic stress has long been investigated as a complex balance between environmental signalling and immunity to modulate plant growth and defence. JA and SA naturally follow a circadian pattern being regulated in antiphase, with JA accumulation peaking at midday during the height of herbivore activity, and SA accumulating at midnight when high humidity and open stomata increase the likelihood of pathogen infection (Zhang *et al*., 2019). This highlights an outstanding question: What role does the circadian clock play in SAR, which is often studied over a period of days? ICS1, a key enzyme in SA biosynthesis, is activated through early‐morning transcription factors, LATE ELONGATED HYPOCOTYL and CIRCADIAN CLOCK ASSOCIATED 1 (CCA1) (Bhardwaj *et al*., 2011) and modulated through transcriptional repression by CCA1 HIKING EXPEDITION (CHE) later in the day (Zhang *et al*., 2019). By contrast, TIME FOR COFFEE (TIC) negatively regulates JA levels through inhibition of MYC2 activity, thereby gating JA responsiveness to optimal times (Shin *et al*., 2012). Interestingly CHE, which regulates *CCA1* expression (Pruneda‐Paz *et al*., 2009), is also known as TCP21, a member of the jasmonate‐responsive transcription factors of the TEOSINTE BRANCHED1/CYCLOIDEA/PROLIFERATING CELL FACTOR1 class. During virus infection, repression of *TCP21* expression via induction of miR319 leads to suppression of JA‐mediated defences and increased susceptibility to *Rice ragged stunt virus* (Zhang *et al*., 2016). CHE is ubiquitylated in the dark, through direct interaction with F‐box‐type E3 ubiquitin ligases ZEITLUPE via its LOV domain (Lee *et al*., 2018). Most interestingly, CHE was recently reported to undergo sulfenylation by RBOHD‐derived H_2_O_2_, enabling binding to the *ICS1* promoter and activating SA production in local systemic tissue (Cao *et al*., 2024; Ikram *et al*., 2025).

Differences in SAR activation are not modulated by circadian rhythms alone, with light exposure duration and quality strongly impacting defence strength. In the dark, failure to accumulate substantial levels of SA and phenylpropanoid enzyme phenylalanine‐ammonia lyase in response to avirulent *Pseudomonas syringae* pv *maculicola* (*Psm*) carrying *avrRpm1* has been reported (Zeier *et al*., 2004), whereas production of JA and camalexin was unaffected (Griebel & Zeier, 2008). Photoreceptor signalling in response to light regulation has markedly influenced SAR timing and intensity, with stronger HR activation, SA production, and pathogenesis‐related gene expression occurring at high light in the morning rather than low light at night following avirulent *Psm* treatment (Griebel & Zeier, 2008) or DC3000*avrRpt2* (Genoud *et al*., 2002) challenges. While loss of UV/blue‐light perception in *Cryptochrome 1 and 2* and *Phototropin 1 and 2* did not significantly impact SAR, reduced phytochrome photo‐perception in the red/far‐red light (RL/FRL) receptor mutant *phyAphyB* resulted in reduced SA and *FMO1* expression and a deficient SAR response (Griebel & Zeier, 2008). However, the requirement for light can extend beyond the synthesis of chemical defences. Chandra‐Shekara *et al*. (2006) demonstrated that light signalling was a prerequisite for the downstream activation of the CNL, HRT, following Turnip Crinkle Virus infection but not for the associated biogenesis of SA. HRT‐mediated resistance required the blue‐light receptors CRY2 and PHOT2 (Jeong *et al*., 2010) and was mediated by the E3 ubiquitin ligase CONSTITUTIVE PHOTOMORPHOGENIC 1 (COP1), which acted as a positive regulator of both HRT and RPM1 stability (Jeong *et al*., 2010; Lim *et al*., 2018). COP1 contributes to R protein stability by safeguarding double‐stranded RNA binding (DRB) proteins, specifically DRB1 and DRB4, whereas the multi‐subunit E3 ligase anaphase‐promoting complex 10 acts as a negative regulator of DRB4 and Turnip Crinkle Virus resistance (Lim *et al*., 2018).

Interestingly, Phytochrome A has also been shown to directly interact with JA synthetase FAR‐RED INSENSITIVE 219 (FIN219) (Jiang *et al*., 2023). FIN219 is also known as JASMONOYL – L‐AMINO ACID SYNTHETASE 1 (JAR1), the enzyme catalysing the formation of the key bioactive jasmonate signal, JA‐Ile. PhyB has been shown to positively regulate defence in tomato against *Spodoptera eridania* (Xiang *et al*., 2022) and tobacco against Cucumber Mottle Virus (Li *et al*., 2015), through activation of downstream JA‐, SA‐, and ET‐defence genes. During the shade‐avoidance response, JA defences are suppressed through PhyB inactivation, which stabilises JAZ repressors and enhances degradation of basic helix–loop–helix (bHLH) MYC transcription factors, reducing sensitivity of JA for defence (Chico *et al*., 2014; Xiang *et al*., 2022). Interestingly, in the context of SAR signalling mechanisms discussed previously, infection of root‐knot nematode triggers GLR3.5‐dependent electrical and Ca^2+^ signals into tomato shoots, where light activates PhyB‐dependent accumulation of ELONGATED HYPOCOTYL 5 (HY5) (Sun *et al*., 2025). These electric signals promote interaction between HY5 and CALMODULIN 2, amplifying activity of HY5 and inducing transcriptional activation of key JA biosynthesis genes. While HY5 acts locally in the leaves, it was also found to move from shoot tissue to the roots, enhancing *GLR3.5* expression and maintaining a reciprocal SAR signalling loop to enhance defence (Sun *et al*., 2025). Obviously, SAR can also be impacted by environmental conditions. A recent paper using the Arabidopsis *PSEUDO‐RESPONSE REGULATOR, prr7/prr9* mutant (whose genetic clock period lengthens to 32 h at 22°C), showed that ETI triggered HR was stronger in the morning but abolished by redox perturbations, as measured by oxidised glutathione (Karapetyan *et al*., 2025). Relevant to the theme of this review, this redox rhythm was gated via JA/ET hormone signalling, not the SA/NPR1 pathway that the genetic clock uses, with chloroplasts and mitochondria making major contributions to this redox reprogramming.

Thus, the environmental impact on plant immunity is complex, occurring at the levels of circadian, redox rhythms, and phytochromes. This emphasises the importance for comparative studies of measuring SAR under consistent conditions including well‐defined infection/inoculum, light intensity and quality, temperature, and daylength parameters, but also highlights that rapidly changing environmental conditions are playing a key role in shaping plant–pathogen immune responses in the field.

## X. Conclusions and future directions

This review distilled recent direct and indirect evidence, which provides a compelling argument that jasmonates play a previously neglected role in plant systemic immunity, both in signal biogenesis, translocation, and likely activation of host immunity in distal responding tissues. By combining historical data with a range of genetic, imaging and spatial transcriptomics data we illustrate the importance of understanding temporal–spatial dynamics and cell state in initiation and establishment of SAR. By its very ability to confer broad spectrum resistance to biotic challenge from herbivory, bacterial, fungal, viral, and oomycete pathogens, SAR must be robust and is unlikely to be elaborated by a single or indeed few signalling molecules alone. Many classical SAR elicitors are themselves inducible, notably SA and NHP, and it is clear that both activation of basal immunity conferred by diverse PRRs as well as ETI elicited by plant disease resistance proteins need to be considered in local responding leaves. Knowledge of SAR signal translocation and systemic decoding remains relatively embryonic, but jasmonates, Ca^2+^, ROS, and GLRs appear to synergise in long distance signalling. Understanding the temporal spatial dynamics of the experimental pathosystem is critically important. A future challenge is determining whether, when and how bifurcation of SAR signalling pathways occurs. Application of fluorescent reporters for jasmonate and SA signalling have recently defined specific cell states demonstrating spatial separation during ETI elicitation and provide an explanation for the co‐existence of SA and JA signalling in ETI. The future will see greater deployment of such imaging approaches in untangling SAR signalling. At the whole plant level, luciferase‐based reporters provide invaluable information for imaging spatial temporal dynamics of SAR and will benefit from new substrate free luciferase lines (Balakireva *et al*., 2026). Such data are critical for informing destructive sampling approaches such as hormone profiling. Building on the importance of cell states, there currently exists a wealth of genetically encoded reporters, capturing glutathione, Ca^2+^, glutamate, SA, and H_2_O_2_ changes, to name a few, in different cellular locations (Wagner & Meyer, 2025). These can provide fine‐scale small molecule resolution to support and help interpret accompanying spatial transcriptomics. Mutant analysis in SAR is challenging as one needs to understand contributions of components in both local and systemic responding tissues. To this end, some signals will be amenable to having inducible reporters in biosynthetic or receptor mutant backgrounds, as elegantly demonstrated in establishing a role for eNAD in SAR. However, an area that has been largely neglected is validating the ‘broad‐spectrum’ nature of SAR. Although challenging, it is important this is addressed in diverse, but experimentally amenable pathosystems. In conclusion, understanding the complexity of SAR will benefit from the deployment of new imaging and ‘omic’ technologies in well‐defined systems, while also extending experimental design to better understand how common environment perturbations, such as heat, drought and light quality, play a role in SAR and disease outcome. We anticipate these will in turn provide new leads for bioengineering inducible broad‐spectrum resistance in crops.

## Competing interests

None declared.

## Author contributions

MG, FB, ES and PK conceptualised, interpreted relevant literature and designed the review structure. ES, MG and FB integrated and analysed data. FB and ES created the figures. All provided insights into the diverse mechanisms impacting plant systemic immune responses and contributed to writing. FB and ES contributed equally to this work.

## Disclaimer

The New Phytologist Foundation remains neutral with regard to jurisdictional claims in maps and in any institutional affiliations.

## References

[nph71405-bib-0001] Aerts N , Pereira Mendes M , Van Wees SCM . 2021. Multiple levels of crosstalk in hormone networks regulating plant defense. The Plant Journal 105: 489–504. 33617121 10.1111/tpj.15124 PMC7898868

[nph71405-bib-0002] Ali Q , Khan AR , Raza W , Bilal MS , Khalid S , Ayaz M , Khan A‐U‐R , Mundra S . 2025. Mechanisms of microbial VOC‐mediated communication in plant ecosystems and agricultural applications. Journal of Sustainable Agriculture and Environment 4: e70044.

[nph71405-bib-0003] Alström S . 1991. Induction of disease resistance in common bean susceptible to halo blight bacterial pathogen after seed bacterization with rhizosphere pseudomonads. The Journal of General and Applied Microbiology 37: 495–501.

[nph71405-bib-0004] Andersson MX , Hamberg M , Kourtchenko O , Brunnström A , KL MP , Gerwick WH , Göbel C , Feussner I , Ellerström M . 2006. Oxylipin profiling of the hypersensitive response in *Arabidopsis thaliana*. Formation of a novel oxo‐phytodienoic acid‐containing galactolipid, arabidopside E. The Journal of Biological Chemistry 281: 31528–31537. 16923817 10.1074/jbc.M604820200

[nph71405-bib-0005] Babikova Z , Gilbert L , Bruce TJ , Birkett M , Caulfield JC , Woodcock C , Pickett JA , Johnson D . 2013. Underground signals carried through common mycelial networks warn neighbouring plants of aphid attack. Ecology Letters 16: 835–843. 23656527 10.1111/ele.12115

[nph71405-bib-0006] Balakireva AV , Karataeva TA , Karampelias M , Mitiouchkina TY , Morozov VV , Macháček J , Shakhova ES , Perfilov MM , Belozerova OA , Kovalchuk SI *et al*. 2026. Non‐invasive imaging of defence responses in plants. Nature Communications. doi: 10.1038/s41467-026-70075-1. PMC13376918 41826305

[nph71405-bib-0007] Barbosa‐Caro JC , Wudick MM . 2024. Revisiting plant electric signaling: challenging an old phenomenon with novel discoveries. Current Opinion in Plant Biology 79: 102528. 38552341 10.1016/j.pbi.2024.102528

[nph71405-bib-0008] Bauer S , Mekonnen DW , Hartmann M , Yildiz I , Janowski R , Lange B , Geist B , Zeier J , Schäffner AR . 2021. UGT76B1, a promiscuous hub of small molecule‐based immune signaling, glucosylates N‐hydroxypipecolic acid, and balances plant immunity. Plant Cell 33: 714–734. 33955482 10.1093/plcell/koaa044 PMC8136890

[nph71405-bib-0009] Bellandi A , Papp D , Breakspear A , Joyce J , Johnston MG , de Keijzer J , Raven EC , Ohtsu M , Vincent TR , Miller AJ *et al*. 2022. Diffusion and bulk flow of amino acids mediate calcium waves in plants. Science Advances 8: eabo6693. 36269836 10.1126/sciadv.abo6693 PMC9586480

[nph71405-bib-0010] Bennett M , Mehta M , Grant M . 2005. Biophoton imaging: a nondestructive method for assaying R gene responses. Molecular Plant Microbe Interactions 18: 95–102. 15720077 10.1094/MPMI-18-0095

[nph71405-bib-0011] Betsuyaku S , Katou S , Takebayashi Y , Sakakibara H , Nomura N , Fukuda H . 2018. Salicylic acid and jasmonic acid pathways are activated in spatially different domains around the infection site during effector‐triggered immunity in *Arabidopsis thaliana* . Plant and Cell Physiology 59: 8–16. 29177423 10.1093/pcp/pcx181 PMC6012717

[nph71405-bib-0012] Bhardwaj V , Meier S , Petersen LN , Ingle RA , Roden LC . 2011. Defence responses of *Arabidopsis thaliana* to infection by *Pseudomonas syringae* are regulated by the circadian clock. PLoS ONE 6: e26968. 22066021 10.1371/journal.pone.0026968 PMC3205005

[nph71405-bib-0148] Birtic S , Ksas B , Genty B , Mueller MJ , Triantaphylidès C , Havaux M . 2011. Using spontaneous photon emission to image lipid oxidation patterns in plant tissues. The Plant Journal 67: 1103–1115. 21595761 10.1111/j.1365-313X.2011.04646.x

[nph71405-bib-0013] Bjornson M , Pimprikar P , Nürnberger T , Zipfel C . 2021. The transcriptional landscape of *Arabidopsis thaliana* pattern‐triggered immunity. Nature Plants 7: 579–586. 33723429 10.1038/s41477-021-00874-5 PMC7610817

[nph71405-bib-0014] Boorboori MR , Lackóová L . 2025. Arbuscular mycorrhizal fungi and salinity stress mitigation in plants. Frontiers in Plant Science 15: 1208. 10.3389/fpls.2024.1504970 PMC11782229 39898265

[nph71405-bib-0015] Brosset A , Blande JD . 2022. Volatile‐mediated plant–plant interactions: volatile organic compounds as modulators of receiver plant defence, growth, and reproduction. Journal of Experimental Botany 73: 511–528. 34791168 10.1093/jxb/erab487 PMC8757495

[nph71405-bib-0016] Brundrett MC , Tedersoo L . 2018. Evolutionary history of mycorrhizal symbioses and global host plant diversity. New Phytologist 220: 1108–1115. 29355963 10.1111/nph.14976

[nph71405-bib-0017] Calabria J , Wang L , Rast‐Somssich MI , Chen HW , Watt M , Persson S , Andersen TG , Idnurm A , Somssich M . 2025. Resolving spatially distinct phytohormone response zones in *Arabidopsis thaliana* roots colonized by Fusarium oxysporum. Journal of Experimental Botany 76: 2022–2034. 39823275 10.1093/jxb/erae516 PMC12066115

[nph71405-bib-0018] Cao H , Bowling SA , Gordon AS , Dong X . 1994. Characterization of an Arabidopsis mutant that is nonresponsive to inducers of systemic acquired resistance. Plant Cell 6: 1583–1592. 12244227 10.1105/tpc.6.11.1583 PMC160545

[nph71405-bib-0019] Cao L , Karapetyan S , Yoo H , Chen T , Mwimba M , Zhang X , Dong X . 2024. H_2_O_2_ sulfenylates CHE, linking local infection to the establishment of systemic acquired resistance. Science 385: 1211–1217. 39265009 10.1126/science.adj7249 PMC11586058

[nph71405-bib-0020] Chanda B , Xia Y , Mandal MK , Yu K , Sekine KT , Gao QM , Selote D , Hu Y , Stromberg A , Navarre D *et al*. 2011. Glycerol‐3‐phosphate is a critical mobile inducer of systemic immunity in plants. Nature Genetics 43: 421–427. 21441932 10.1038/ng.798

[nph71405-bib-0021] Chandra‐Shekara AC , Gupte M , Navarre D , Raina S , Raina R , Klessig D , Kachroo P . 2006. Light‐dependent hypersensitive response and resistance signaling against Turnip Crinkle Virus in Arabidopsis. The Plant Journal 45: 320–334. 16412080 10.1111/j.1365-313X.2005.02618.x

[nph71405-bib-0022] Chaturvedi R , Venables B , Petros RA , Nalam V , Li M , Wang X , Takemoto LJ , Shah J . 2012. An abietane diterpenoid is a potent activator of systemic acquired resistance. The Plant Journal 71: 161–172. 22385469 10.1111/j.1365-313X.2012.04981.x

[nph71405-bib-0023] Chester KS . 1933. The problem of acquired physiological immunity in plants. The Quarterly Review of Biology 8: 129–154.

[nph71405-bib-0024] Chico J‐M , Fernández‐Barbero G , Chini A , Fernández‐Calvo P , Díez‐Díaz M , Solano R . 2014. Repression of jasmonate‐dependent defenses by shade involves differential regulation of protein stability of MYC transcription factors and their JAZ repressors in Arabidopsis. Plant Cell 26: 1967–1980. 24824488 10.1105/tpc.114.125047 PMC4079362

[nph71405-bib-0025] Cofer TM , Engelberth M , Engelberth J . 2018. Green leaf volatiles protect maize (*Zea mays*) seedlings against damage from cold stress. Plant, Cell & Environment 41: 1673–1682. 10.1111/pce.13204 29601632

[nph71405-bib-0026] Conrath U . 2025. Cross‐kingdom mechanisms of trained immunity in plant systemic acquired resistance. Nature Plants 11: 1993–2005. 41028451 10.1038/s41477-025-02119-1

[nph71405-bib-0027] Cui J , Bahrami AK , Pringle EG , Hernandez‐Guzman G , Bender CL , Pierce NE , Ausubel FM . 2005. *Pseudomonas syringae* manipulates systemic plant defenses against pathogens and herbivores. Proceedings of the National Academy of Sciences, USA 102: 1791–1796. 10.1073/pnas.0409450102 PMC547856 15657122

[nph71405-bib-0028] Ding Y , Sun T , Ao K , Peng Y , Zhang Y , Li X , Zhang Y . 2018. Opposite roles of salicylic acid receptors NPR1 and NPR3/NPR4 in transcriptional regulation of plant immunity. Cell 173: 1454–1467. 29656896 10.1016/j.cell.2018.03.044

[nph71405-bib-0029] Dmitrieva VA , Tyutereva EV , Voitsekhovskaja OV . 2020. Singlet oxygen in plants: generation, detection, and signaling roles. International Journal of Molecular Sciences 21: 426. 32375245 10.3390/ijms21093237 PMC7247340

[nph71405-bib-0030] Farmer EE , Gasperini D , Acosta IF . 2014. The squeeze cell hypothesis for the activation of jasmonate synthesis in response to wounding. New Phytologist 204: 282–288. 25453132 10.1111/nph.12897

[nph71405-bib-0031] Forczek ST , Bukovská P , Püschel D , Janoušková M , Blažková A , Jansa J . 2022. Drought rearranges preferential carbon allocation to arbuscular mycorrhizal community members co‐inhabiting roots of *Medicago truncatula* . Environmental and Experimental Botany 199: 104897.

[nph71405-bib-0032] Frank L , Wenig M , Ghirardo A , van der Krol A , Vlot AC , Schnitzler J‐P , Rosenkranz M . 2021. Isoprene and β‐caryophyllene confer plant resistance via different plant internal signalling pathways. Plant, Cell & Environment 44: 1151–1164. 10.1111/pce.14010 33522606

[nph71405-bib-0033] Frost CJ , Appel HM , Carlson JE , De Moraes CM , Mescher MC , Schultz JC . 2007. Within‐plant signalling via volatiles overcomes vascular constraints on systemic signalling and primes responses against herbivores. Ecology Letters 10: 490–498. 17498148 10.1111/j.1461-0248.2007.01043.x

[nph71405-bib-0034] Fuentes S , Tongson E , Unnithan RR , Gonzalez Viejo C . 2021. Early detection of aphid infestation and insect‐plant interaction assessment in wheat using a low‐cost electronic nose (e‐nose), near‐infrared spectroscopy and machine learning modeling. Sensors 21: 5948. 34502839 10.3390/s21175948 PMC8434653

[nph71405-bib-0035] Gaikwad T , Breen S , Breeze E , Stroud E , Hussain R , Kulasekaran S , Kargios N , Bennett F , de Torres‐Zabala M , Horsell D *et al*. 2026. Rapid local and systemic jasmonate signalling drives the initiation and establishment of plant systemic immunity. Nature Plants 12: 152–163. 41495459 10.1038/s41477-025-02178-4 PMC12830360

[nph71405-bib-0036] Gao Y‐Q , Jimenez‐Sandoval P , Tiwari S , Stolz S , Wang J , Glauser G , Santiago J , Farmer EE . 2023. Ricca's factors as mobile proteinaceous effectors of electrical signaling. Cell 186: 1337–1351.e1320. 36870332 10.1016/j.cell.2023.02.006 PMC10098372

[nph71405-bib-0037] Genoud T , Buchala AJ , Chua N‐H , Métraux J‐P . 2002. Phytochrome signalling modulates the SA‐perceptive pathway in Arabidopsis. The Plant Journal 31: 87–95. 12100485 10.1046/j.1365-313x.2002.01338.x

[nph71405-bib-0038] Gilroy S . 2025. Waving hello to pathogens: the dynamics of the spread of plant MAMP‐triggered Ca^2+^ signaling. Science Signaling 18: eaeb7956. 41329791 10.1126/scisignal.aeb7956

[nph71405-bib-0039] Grant M , Brown I , Adams S , Knight M , Ainslie A , Mansfield J . 2000. The RPM1 plant disease resistance gene facilitates a rapid and sustained increase in cytosolic calcium that is necessary for the oxidative burst and hypersensitive cell death. The Plant Journal 23: 441–450. 10972870 10.1046/j.1365-313x.2000.00804.x

[nph71405-bib-0040] Grenzi M , Bonza MC , Alfieri A , Costa A . 2021. Structural insights into long‐distance signal transduction pathways mediated by plant glutamate receptor‐like channels. New Phytologist 229: 1261–1267. 33107608 10.1111/nph.17034

[nph71405-bib-0041] Griebel T , Zeier J . 2008. Light regulation and daytime dependency of inducible plant defenses in arabidopsis: phytochrome signaling controls systemic acquired resistance rather than local defense. Plant Physiology 147: 790–801. 18434604 10.1104/pp.108.119503 PMC2409012

[nph71405-bib-0042] Hartmann M , Zeier T , Bernsdorff F , Reichel‐Deland V , Kim D , Hohmann M , Scholten N , Schuck S , Bräutigam A , Hölzel T *et al*. 2018. Flavin monooxygenase‐generated N‐hydroxypipecolic acid is a critical element of plant systemic immunity. Cell 173: 456–469. 29576453 10.1016/j.cell.2018.02.049

[nph71405-bib-0043] Hou S , Liu Z , Shen H , Wu D . 2019. Damage‐associated molecular pattern‐triggered immunity in plants. Frontiers in Plant Science 10: 12019. 10.3389/fpls.2019.00646 PMC6547358 31191574

[nph71405-bib-0044] Hou S , Tsuda K . 2022. Salicylic acid and jasmonic acid crosstalk in plant immunity. Essays in Biochemistry 66: 647–656. 35698792 10.1042/EBC20210090

[nph71405-bib-0045] Ibrahim T , King FJ , Toghani A , Wang L , Jenkins S , Yuen ELH , Wang H‐Y , Vuolo C , Eilmann N , Adamkova V *et al*. 2026. A helper NLR channels organellar calcium to trigger plant immunity. Science 392: 499–505. 42060733 10.1126/science.aeb6690

[nph71405-bib-0046] Ikram AU , Chen H , Chen J . 2025. H_2_O_2_ sulfenylates CHE to activate systemic acquired resistance. Trends in Plant Science 30: 238–240. 39743404 10.1016/j.tplants.2024.12.007

[nph71405-bib-0047] Ilham B , Noureddine C , Philippe G , Mohammed EG , Brahim E , Sophie A , Martine N , Muriel M . 2019. Induced systemic resistance (ISR) in *Arabidopsis thaliana* by Bacillus amyloliquefaciens and *Trichoderma harzianum* used as seed treatments. Agriculture 9: 166.

[nph71405-bib-0048] Jacob P , Hige J , Dangl JL . 2023. Is localized acquired resistance the mechanism for effector‐triggered disease resistance in plants? Nature Plants 9: 1184–1190. 37537398 10.1038/s41477-023-01466-1

[nph71405-bib-0049] Jeong RD , Chandra‐Shekara AC , Barman SR , Navarre D , Klessig DF , Kachroo A , Kachroo P . 2010. Cryptochrome 2 and phototropin 2 regulate resistance protein‐mediated viral defense by negatively regulating an E3 ubiquitin ligase. Proceedings of the National Academy of Sciences, USA 107: 13538–13543. 10.1073/pnas.1004529107 PMC2922132 20624951

[nph71405-bib-0050] Jiang H‐W , Peng K‐C , Hsu T‐Y , Chiou Y‐C , Hsieh H‐L . 2023. Arabidopsis FIN219/JAR1 interacts with phytochrome A under far‐red light and jasmonates in regulating hypocotyl elongation via a functional demand manner. PLoS Genetics 19: e1010779. 37216398 10.1371/journal.pgen.1010779 PMC10237651

[nph71405-bib-0051] Jung HW , Tschaplinski TJ , Wang L , Glazebrook J , Greenberg JT . 2009. Priming in systemic plant immunity. Science 324: 89–91. 19342588 10.1126/science.1170025

[nph71405-bib-0052] Kachroo P , Burch‐Smith TM , Grant M . 2021. An emerging role for chloroplasts in disease and defense. Annual Review of Phytopathology 59: 423–445. 10.1146/annurev-phyto-020620-115813 34432508

[nph71405-bib-0053] Samaras A , Roumeliotis E , Ntasiou P , Karaoglanidis G . 2021. *Bacillus subtilis* MBI600 promotes growth of tomato plants and induces systemic resistance contributing to the control of soilborne pathogens. Plants 10: 1113. 34072940 10.3390/plants10061113 PMC8229581

[nph71405-bib-0054] Karapetyan S , Mwimba M , Chen T , Yao Z , Dong X . 2025. The redox rhythm gates immune‐induced cell death distinctly from the genetic clock. Proceedings of the National Academy of Sciences, USA 122: e2519251122. 10.1073/pnas.2519251122 PMC12452835 40928881

[nph71405-bib-0055] Kenton P , Mur LAJ , Atzorn R , Wasternack C , Draper J . 1999. (‐)‐Jasmonic acid accumulation in tobacco hypersensitive response lesions. Molecular Plant Microbe Interactions 12: 74–78.

[nph71405-bib-0056] Keppler LD , Novacky A . 1987. The initiation of membrane lipid peroxidation during bacteria‐induced hypersensitive reaction. Physiological and Molecular Plant Pathology 30: 233–245.

[nph71405-bib-0057] Kim JY , Symeonidi E , Pang TY , Denyer T , Weidauer D , Bezrutczyk M , Miras M , Zöllner N , Hartwig T , Wudick MM *et al*. 2021. Distinct identities of leaf phloem cells revealed by single cell transcriptomics. Plant Cell 33: 511–530. 33955487 10.1093/plcell/koaa060 PMC8136902

[nph71405-bib-0058] Kim N , Chae E , Song J‐J . 2026. Plant NLRs are getting into higher‐order architectures. The Plant Journal 126: e70855. 41968525 10.1111/tpj.70855 PMC13071261

[nph71405-bib-0059] Klessig DF , Choi HW , Dempsey DA . 2018. Systemic acquired resistance and salicylic acid: past, present, and future. Molecular Plant Microbe Interactions 31: 871–888. 29781762 10.1094/MPMI-03-18-0067-CR

[nph71405-bib-0060] Kong HG , Song GC , Sim H‐J , Ryu C‐M . 2021. Achieving similar root microbiota composition in neighbouring plants through airborne signalling. The ISME Journal 15: 397–408. 32973341 10.1038/s41396-020-00759-z PMC8027813

[nph71405-bib-0061] Köster P , DeFalco TA , Zipfel C . 2022. Ca^2+^ signals in plant immunity. EMBO Journal 41: EMBJ2022110741. 10.15252/embj.2022110741 PMC9194748 35560235

[nph71405-bib-0062] Kumari A , Chételat A , Nguyen CT , Farmer EE . 2019. Arabidopsis H(+)‐ATPase AHA1 controls slow wave potential duration and wound‐response jasmonate pathway activation. Proceedings of the National Academy of Sciences, USA 116: 20226–20231. 10.1073/pnas.1907379116 PMC6778210 31527254

[nph71405-bib-0063] Kundu P , Kumari M , Meena MK , Mishra S , Vadassery J . 2025. The Arabidopsis eATP receptor DORN1 and CNGC19 calcium channel act in tandem to regulate plant defense upon *Spodoptera litura* herbivory. Journal of Experimental Botany 76: 2264–2277. 39862227 10.1093/jxb/eraf025

[nph71405-bib-0064] Lee C‐M , Feke A , Li M‐W , Adamchek C , Webb K , Pruneda‐Paz J , Bennett EJ , Kay SA , Gendron JM . 2018. Decoys untangle complicated redundancy and reveal targets of circadian clock F‐box proteins. Plant Physiology 177: 1170–1186. 29794020 10.1104/pp.18.00331 PMC6052990

[nph71405-bib-0065] Levak V , Povalej TM , Pogačar K , Stare K , Zagorščak M , Hawkins T , Robson J , Dobnik D , Lukan T , Gruden K . 2025. Jasmonic acid and salicylic acid interact to determine spatial regulation of gene expression responses in potato leaf to herbivory by Colorado potato beetle and mechanical wounding. Plant & Cell Physiology 66: 1878–1890. 40990354 10.1093/pcp/pcaf120 PMC12739108

[nph71405-bib-0066] Li J‐Y , Deng X‐G , Chen L‐J , Fu F‐Q , Pu X‐J , Xi D‐H , Lin H‐H . 2015. Involvement of PHYB in resistance to Cucumber mosaic virus in *Nicotiana tabacum* . Plant Growth Regulation 77: 33–42.

[nph71405-bib-0067] Li M , Wang F , Li S , Yu G , Wang L , Li Q , Zhu X , Li Z , Yuan L , Liu P . 2020. Importers drive leaf‐to‐leaf jasmonic acid transmission in wound‐induced systemic immunity. Molecular Plant 13: 1485–1498. 32889174 10.1016/j.molp.2020.08.017

[nph71405-bib-0068] Li Q , Zhou M , Chhajed S , Yu F , Chen S , Zhang Y , Mou Z . 2023. N‐hydroxypipecolic acid triggers systemic acquired resistance through extracellular NAD(P). Nature Communications 14: 6848. 10.1038/s41467-023-42629-0 PMC10611778 37891163

[nph71405-bib-0069] Li X , Sung Y‐C , Coaker G , Zhu J . 2026. Spatial organization of plant defense at the infection front. bioRxiv. doi: 10.64898/2026.04.01.715938.

[nph71405-bib-0070] Lim G‐H , Hoey T , Zhu S , Clavel M , Yu K , Navarre D , Kachroo A , Deragon J‐M , Kachroo P . 2018. COP1, a negative regulator of photomorphogenesis, positively regulates plant disease resistance via double‐stranded RNA binding proteins. PLoS Pathogens 14: e1006894. 29513740 10.1371/journal.ppat.1006894 PMC5871017

[nph71405-bib-0071] Lim GH , Liu H , Yu K , Liu R , Shine MB , Fernandez J , Burch‐Smith T , Mobley JK , McLetchie N , Kachroo A *et al*. 2020. The plant cuticle regulates apoplastic transport of salicylic acid during systemic acquired resistance. Science Advances 6: eaaz0478. 32494705 10.1126/sciadv.aaz0478 PMC7202870

[nph71405-bib-0072] Littlejohn GR , Breen S , Smirnoff N , Grant M . 2021. Chloroplast immunity illuminated. New Phytologist 229: 3088–3107. 33206379 10.1111/nph.17076

[nph71405-bib-0073] van Loon LC , Bakker PAHM , Pieterse CMJ . 1998. Systemic resistance induced by rhizosphere bacteria. Annual Review of Phytopathology 36: 453–483. 10.1146/annurev.phyto.36.1.453 15012509

[nph71405-bib-0074] Ma X , Denyer T , Timmermans MCP . 2020. pscb: a browser to explore plant single cell RNA‐sequencing data sets. Plant Physiology 183: 464–467. 32209591 10.1104/pp.20.00250 PMC7271789

[nph71405-bib-0075] Manzoor H , Kelloniemi J , Chiltz A , Wendehenne D , Pugin A , Poinssot B , Garcia‐Brugger A . 2013. Involvement of the glutamate receptor AtGLR3.3 in plant defense signaling and resistance to Hyaloperonospora arabidopsidis. The Plant Journal 76: 466–480. 23952652 10.1111/tpj.12311

[nph71405-bib-0076] Martel A , Ruiz‐Bedoya T , Breit‐McNally C , Laflamme B , Desveaux D , Guttman DS . 2021. The ETS‐ETI cycle: evolutionary processes and metapopulation dynamics driving the diversification of pathogen effectors and host immune factors. Current Opinion in Plant Biology 62: 102011. 33677388 10.1016/j.pbi.2021.102011

[nph71405-bib-0077] Martin R , Qi T , Zhang H , Liu F , King M , Toth C , Nogales E , Staskawicz BJ . 2020. Structure of the activated ROQ1 resistosome directly recognizing the pathogen effector XopQ. Science 370: eabd9993. 33273074 10.1126/science.abd9993 PMC7995448

[nph71405-bib-0078] Maruta N , Sorbello M , Garzon‐Flores L , Kobe B . 2026. Molecular mechanisms of plant NLR activation and signalling. The Plant Journal 125: e70702. 41614442 10.1111/tpj.70702 PMC12856790

[nph71405-bib-0079] Meyerhoff O , Müller K , Roelfsema MR , Latz A , Lacombe B , Hedrich R , Dietrich P , Becker D . 2005. AtGLR3.4, a glutamate receptor channel‐like gene is sensitive to touch and cold. Planta 222: 418–427. 15864638 10.1007/s00425-005-1551-3

[nph71405-bib-0080] Mineiro M , Groux R , Gouhier‐Darimont C , Mateo P , Robert CAM , Reymond P . 2025. The glutamate receptor‐like GLR2.7 modulates insect egg‐induced defense responses in Arabidopsis. New Phytologist 248: 897–912. 40716030 10.1111/nph.70405 PMC12445815

[nph71405-bib-0081] Mishina TE , Zeier J . 2007. Pathogen‐associated molecular pattern recognition rather than development of tissue necrosis contributes to bacterial induction of systemic acquired resistance in Arabidopsis. The Plant Journal 50: 500–513. 17419843 10.1111/j.1365-313X.2007.03067.x

[nph71405-bib-0082] Mohnike L , Rekhter D , Huang W , Feussner K , Tian H , Herrfurth C , Zhang Y , Feussner I . 2021. The glycosyltransferase UGT76B1 modulates N‐hydroxy‐pipecolic acid homeostasis and plant immunity. Plant Cell 33: 735–749. 33955489 10.1093/plcell/koaa045 PMC8136917

[nph71405-bib-0083] Morin H , Chételat A , Stolz S , Marcourt L , Glauser G , Wolfender J‐L , Farmer EE . 2023. Wound‐response jasmonate dynamics in the primary vasculature. New Phytologist 240: 1484–1496. 37598308 10.1111/nph.19207

[nph71405-bib-0084] Mousavi SAR , Chauvin A , Pascaud F , Kellenberger S , Farmer EE . 2013. GLUTAMATE RECEPTOR‐LIKE genes mediate leaf‐to‐leaf wound signalling. Nature 500: 422–426. 23969459 10.1038/nature12478

[nph71405-bib-0085] Návarová H , Bernsdorff F , Döring AC , Zeier J . 2012. Pipecolic acid, an endogenous mediator of defense amplification and priming, is a critical regulator of inducible plant immunity. Plant Cell 24: 5123–5141. 23221596 10.1105/tpc.112.103564 PMC3556979

[nph71405-bib-0086] Nguyen CT , Kurenda A , Stolz S , Chételat A , Farmer EE . 2018. Identification of cell populations necessary for leaf‐to‐leaf electrical signaling in a wounded plant. Proceedings of the National Academy of Sciences, USA 115(40): 10178–10183. 10.1073/pnas.1807049115 PMC6176584 30228123

[nph71405-bib-0087] Nobori T . 2025. Immune cell states: critical building blocks of the plant immune system. Cell Host & Microbe 33: 1227–1232. 40812174 10.1016/j.chom.2025.06.016

[nph71405-bib-0088] Nobori T , Monell A , Lee TA , Sakata Y , Shirahama S , Zhou J , Nery JR , Mine A , Ecker JR . 2025. A rare PRIMER cell state in plant immunity. Nature 638: 197–205. 39779856 10.1038/s41586-024-08383-z PMC11798839

[nph71405-bib-0089] Patel HK , Gomes EN , Wu Q , Patel N , Kobayashi DY , Wang C , Simon JE . 2023. Volatile metabolites from new cultivars of catnip and oregano as potential antibacterial and insect repellent agents. Frontiers in Plant Science 14: 24305. 10.3389/fpls.2023.1124305 PMC9995836 36909430

[nph71405-bib-0090] Peer RV , Niemann GJ , Schippers B . 1991. Induced resistance and phytoalexin accumulation in biological control of Fusarium wilt of carnation by *Pseudomonas* sp. strain WCS417r. Phytopathology 81: 728–734.

[nph71405-bib-0091] Pieterse CM , van Wees SC , Hoffland E , van Pelt JA , van Loon LC . 1996. Systemic resistance in Arabidopsis induced by biocontrol bacteria is independent of salicylic acid accumulation and pathogenesis‐related gene expression. Plant Cell 8: 1225–1237. 8776893 10.1105/tpc.8.8.1225 PMC161233

[nph71405-bib-0092] Pieterse CMJ , van Wees SCM , van Pelt JA , Knoester M , Laan R , Gerrits H , Weisbeek PJ , van Loon LC . 1998. A novel signaling pathway controlling induced systemic resistance in Arabidopsis. Plant Cell 10: 1571–1580. 9724702 10.1105/tpc.10.9.1571 PMC144073

[nph71405-bib-0093] Polwatte Koralalage DS , Wang Q , Mohotti J , Zhang L , Li C , Chang M . 2026. Jasmonate before salicylate in systemic acquired resistance. Trends in Plant Science. doi: 10.1016/j.tplants.2026.03.009. 41963135

[nph71405-bib-0094] Pršić J , Ongena M . 2020. Elicitors of plant immunity triggered by beneficial bacteria. Frontiers in Plant Science 11: 4530. 10.3389/fpls.2020.594530 PMC7693457 33304371

[nph71405-bib-0095] Pruneda‐Paz JL , Breton G , Para A , Kay SA . 2009. A functional genomics approach reveals CHE as a component of the *Arabidopsis* circadian clock. Science 323: 1481–1485. 19286557 10.1126/science.1167206 PMC4259050

[nph71405-bib-0096] Rabari A , Ruparelia J , Jha CK , Sayyed RZ , Mitra D , Priyadarshini A , Senapati A , Panneerselvam P , Das Mohapatra PK . 2023. Articulating beneficial rhizobacteria‐mediated plant defenses through induced systemic resistance: a review. Pedosphere 33: 556–566.

[nph71405-bib-0097] Riedlmeier M , Ghirardo A , Wenig M , Knappe C , Koch K , Georgii E , Dey S , Parker JE , Schnitzler J‐P , Vlot AC . 2017. Monoterpenes support systemic acquired resistance within and between plants. Plant Cell 29: 1440–1459. 28536145 10.1105/tpc.16.00898 PMC5502447

[nph71405-bib-0098] Ross AF . 1961. Systemic acquired resistance induced by localized virus infections in plants. Virology 14: 340–358. 13743578 10.1016/0042-6822(61)90319-1

[nph71405-bib-0099] Roussin‐Léveillée C , St‐Amand M , Desbiens‐Fortin P , Perreault R , Pelletier A , Gauthier S , Gaudreault‐Lafleur F , Laforest‐Lapointe I , Moffett P . 2025. Co‐occurrence of chloroplastic ROS production and salicylic acid induction in plant immunity. New Phytologist 248: 1989–2004. 40948314 10.1111/nph.70569 PMC12529033

[nph71405-bib-0100] Schilmiller AL , Howe GA . 2005. Systemic signaling in the wound response. Current Opinion in Plant Biology 8: 369–377. 15939667 10.1016/j.pbi.2005.05.008

[nph71405-bib-0101] Shin J , Heidrich K , Sanchez‐Villarreal A , Parker JE , Davis SJ . 2012. TIME FOR COFFEE represses accumulation of the MYC2 transcription factor to provide time‐of‐day regulation of jasmonate signaling in Arabidopsis. Plant Cell 24: 2470–2482. 22693280 10.1105/tpc.111.095430 PMC3406923

[nph71405-bib-0102] Shine MB , Zhang K , Liu H , Lim GH , Xia F , Yu K , Hunt AG , Kachroo A , Kachroo P . 2022. Phased small RNA–mediated systemic signaling in plants. Science Advances 8: eabm8791. 35749505 10.1126/sciadv.abm8791 PMC9232115

[nph71405-bib-0103] Shu L‐J , Kahlon PS , Ranf S . 2023. The power of patterns: new insights into pattern‐triggered immunity. New Phytologist 240: 960–967. 37525301 10.1111/nph.19148

[nph71405-bib-0104] Snoeck S , Abramson BW , Garcia AGK , Egan AN , Michael TP , Steinbrenner AD . 2022. Evolutionary gain and loss of a plant pattern‐recognition receptor for HAMP recognition. eLife 11: e81050. 36377784 10.7554/eLife.81050 PMC9718524

[nph71405-bib-0105] Song GC , Ryu CM . 2018. Evidence for volatile memory in plants: boosting defence priming through the recurrent application of plant volatiles. Molecules and Cells 41: 724–732. 29991670 10.14348/molcells.2018.0104 PMC6125420

[nph71405-bib-0106] Song YY , Ye M , Li C , He X , Zhu‐Salzman K , Wang RL , Su YJ , Luo SM , Zeng RS . 2014. Hijacking common mycorrhizal networks for herbivore‐induced defence signal transfer between tomato plants. Scientific Reports 4: 3915. 24468912 10.1038/srep03915 PMC3904153

[nph71405-bib-0107] Song YY , Zeng RS , Xu JF , Li J , Shen X , Yihdego WG . 2010. Interplant communication of tomato plants through underground common mycorrhizal networks. PLoS ONE 5: e13324. 20967206 10.1371/journal.pone.0013324 PMC2954164

[nph71405-bib-0108] Spoel SH , Dong X . 2008. Making sense of hormone crosstalk during plant immune responses. Cell Host & Microbe 3: 348–351. 18541211 10.1016/j.chom.2008.05.009

[nph71405-bib-0109] Spoel SH , Dong X . 2024. Salicylic acid in plant immunity and beyond. Plant Cell 36: 1451–1464. 38163634 10.1093/plcell/koad329 PMC11062473

[nph71405-bib-0110] Spoel SH , Johnson JS , Dong X . 2007. Regulation of tradeoffs between plant defenses against pathogens with different lifestyles. Proceedings of the National Academy of Sciences, USA 104: 18842–18847. 10.1073/pnas.0708139104 PMC2141864 17998535

[nph71405-bib-0111] Stroud EA , Jayaraman J , Templeton MD , Rikkerink EHA . 2022. Comparison of the pathway structures influencing the temporal response of salicylate and jasmonate defence hormones in *Arabidopsis thaliana* . Frontiers in Plant Science 13: 952301. 36160984 10.3389/fpls.2022.952301 PMC9504473

[nph71405-bib-0112] Su F , Zhao B , Dhondt‐Cordelier S , Vaillant‐Gaveau N . 2024. Plant‐growth‐promoting rhizobacteria modulate carbohydrate metabolism in connection with host plant defense mechanism. International Journal of Molecular Sciences 25: 1465. 38338742 10.3390/ijms25031465 PMC10855160

[nph71405-bib-0113] Suda H , Toyota M . 2022. Integration of long‐range signals in plants: a model for wound‐induced Ca^2+^, electrical, ROS, and glutamate waves. Current Opinion in Plant Biology 69: 102270. 35926395 10.1016/j.pbi.2022.102270

[nph71405-bib-0114] Sun L , Wang Y , Zhang J . 2026. Calcium signaling in plant defense. New Plant Protection 3: e70028.

[nph71405-bib-0115] Sun T , Hu C , Zhu C , Cai J , Wang G , Xia X , Shi K , Zhou Y , Yu J , Foyer CH *et al*. 2025. HY5 integrates light and electrical signaling to trigger a jasmonate burst for nematode defense in tomato. Nature Communications 16: 8750. 10.1038/s41467-025-63779-3 PMC12489108 41034216

[nph71405-bib-0116] Thaler JS , Humphrey PT , Whiteman NK . 2012. Evolution of jasmonate and salicylate signal crosstalk. Trends in Plant Science 17: 260–270. 22498450 10.1016/j.tplants.2012.02.010

[nph71405-bib-0117] Toyota M , Spencer D , Sawai‐Toyota S , Jiaqi W , Zhang T , Koo AJ , Howe GA , Gilroy S . 2018. Glutamate triggers long‐distance, calcium‐based plant defense signaling. Science 361: 1112–1115. 30213912 10.1126/science.aat7744

[nph71405-bib-0118] Triantaphylidès C , Krischke M , Hoeberichts FA , Ksas B , Gresser G , Havaux M , Van Breusegem F , Mueller MJ . 2008. Singlet oxygen is the major reactive oxygen species involved in photooxidative damage to plants. Plant Physiology 148: 960–968. 18676660 10.1104/pp.108.125690 PMC2556806

[nph71405-bib-0119] Truman W , Bennett MH , Kubigsteltig I , Turnbull C , Grant M . 2007. *Arabidopsis* systemic immunity uses conserved defense signaling pathways and is mediated by jasmonates. Proceedings of the National Academy of Sciences, USA 104: 1075–1080. 10.1073/pnas.0605423104 PMC1783366 17215350

[nph71405-bib-0120] Van der Ent S , Verhagen BW , Van Doorn R , Bakker D , Verlaan MG , Pel MJ , Joosten RG , Proveniers MC , Van Loon LC , Ton J *et al*. 2008. MYB72 is required in early signaling steps of rhizobacteria‐induced systemic resistance in Arabidopsis. Plant Physiology 146: 1293–1304. 18218967 10.1104/pp.107.113829 PMC2259080

[nph71405-bib-0121] Vlot AC , Sales JH , Lenk M , Bauer K , Brambilla A , Sommer A , Chen Y , Wenig M , Nayem S . 2021. Systemic propagation of immunity in plants. New Phytologist 229: 1234–1250. 32978988 10.1111/nph.16953

[nph71405-bib-0122] Wagner S , Meyer AJ . 2025. Illuminating plant metabolism with genetically encoded biosensors. Journal of Plant Physiology 311: 154498. 40544579 10.1016/j.jplph.2025.154498

[nph71405-bib-0123] Wang C , Huang X , Li Q , Zhang Y , Li JL , Mou Z . 2019. Extracellular pyridine nucleotides trigger plant systemic immunity through a lectin receptor kinase/BAK1 complex. Nature Communications 10: 4810. 10.1038/s41467-019-12781-7 PMC6805918 31641112

[nph71405-bib-0124] Wang H , Li X , Zhai K , Rhodes J , Sang T , Zhao J , Gao Y , Ma S , Song B , Pan Q *et al*. 2026. A regulatory network promotes apoplastic alkalinization to prime plant immunity in tissues distal to site of infection. Cell 189: 1389–1406. 41742413 10.1016/j.cell.2026.01.027

[nph71405-bib-0125] Wang J , Sun X , Xiong F , Lapin D , Lee T , Martin‐Ramirez S , Prakken A , Shen Q , Bautor J , Maekawa T *et al*. 2025. Coordinated actions of NLR‐assembled and glutamate receptor–like calcium channels in plant effector‐triggered immunity. Proceedings of the National Academy of Sciences, USA 122: e2508018122. 10.1073/pnas.2508018122 PMC12415192 40844808

[nph71405-bib-0126] Wang Q‐Q , Cheng N , Yi W‐B , Peng S‐M , Zou X‐Q . 2014. Synthesis, nitric oxide release, and α‐glucosidase inhibition of nitric oxide donating apigenin and chrysin derivatives. Bioorganic & Medicinal Chemistry 22: 1515–1521. 24508143 10.1016/j.bmc.2014.01.038

[nph71405-bib-0127] van Wees SCM , de Swart EAM , van Pelt JA , van Loon LC , Pieterse CMJ . 2000. Enhancement of induced disease resistance by simultaneous activation of salicylate‐ and jasmonate‐dependent defense pathways in *Arabidopsis thaliana* . Proceedings of the National Academy of Sciences, USA 97: 8711–8716. 10.1073/pnas.130425197 PMC27013 10890883

[nph71405-bib-0128] Wei G . 1991. Induction of systemic resistance of cucumber to *Colletotrichum orbiculare* by select strains of plant growth‐promoting rhizobacteria. Phytopathology 81: 1508–1512.

[nph71405-bib-0129] Wenig M , Ghirardo A , Sales JH , Pabst ES , Breitenbach HH , Antritter F , Weber B , Lange B , Lenk M , Cameron RK *et al*. 2019. Systemic acquired resistance networks amplify airborne defense cues. Nature Communications 10: 3813. 10.1038/s41467-019-11798-2 PMC6707303 31444353

[nph71405-bib-0130] Wipf D , Krajinski F , van Tuinen D , Recorbet G , Courty P‐E . 2019. Trading on the arbuscular mycorrhiza market: from arbuscules to common mycorrhizal networks. New Phytologist 223: 1127–1142. 30843207 10.1111/nph.15775

[nph71405-bib-0131] Wu F , Chi Y , Jiang Z , Xu Y , Xie L , Huang F , Wan D , Ni J , Yuan F , Wu X *et al*. 2020. Hydrogen peroxide sensor HPCA1 is an LRR receptor kinase in Arabidopsis. Nature 578: 577–581. 32076270 10.1038/s41586-020-2032-3

[nph71405-bib-0132] Xia F , Liu H , Yu K , Yuan X , Kachroo A , Kachroo P . 2026. Excess nitric oxide alters cellular pH to restrict salicylic acid movement and systemic immunity. Science Advances 12: 1268. 10.1126/sciadv.adz4776 PMC13220883 42213848

[nph71405-bib-0133] Xiang S , Wu S , Jing Y , Chen L , Yu D . 2022. Phytochrome B regulates jasmonic acid‐mediated defense response against *Botrytis cinerea* in Arabidopsis. Plant Diversity 44: 109–115. 35281129 10.1016/j.pld.2021.01.007 PMC8897165

[nph71405-bib-0134] Xu P , Fundneider S , Lange B , Maksym R , Stuttmann J , Schäffner AR . 2025. A root‐based N‐hydroxypipecolic acid standby circuit to direct immunity and growth of Arabidopsis shoots. Nature Plants 11: 1658–1669. 40696005 10.1038/s41477-025-02053-2 PMC12364709

[nph71405-bib-0135] Yan Y , Liu G , Shen Q , He P , Shan L . 2025. Orchestration of plant PRR‐ and NLR‐mediated immunity: Protein kinases and beyond. Molecular Cell 85: 3840–3852. 41106372 10.1016/j.molcel.2025.09.010 PMC12614328

[nph71405-bib-0136] Yi H‐S , Heil M , Adame‐Álvarez RM , Ballhorn DJ , Ryu C‐M . 2009. Airborne induction and priming of plant defenses against a bacterial pathogen. Plant Physiology 151: 2152–2161. 19812184 10.1104/pp.109.144782 PMC2785983

[nph71405-bib-0137] Yildiz I , Mantz M , Hartmann M , Zeier T , Kessel J , Thurow C , Gatz C , Petzsch P , Köhrer K , Zeier J . 2021. The mobile SAR signal N‐hydroxypipecolic acid induces NPR1‐dependent transcriptional reprogramming and immune priming. Plant Physiology 186: 1679–1705. 33871649 10.1093/plphys/kiab166 PMC8260123

[nph71405-bib-0138] Yu Y , Gui Y , Li Z , Jiang C , Guo J , Niu D . 2022. Induced systemic resistance for improving plant immunity by beneficial microbes. Plants 11: 386. 35161366 10.3390/plants11030386 PMC8839143

[nph71405-bib-0139] Yuan M , Huang Y , Ge W , Jia Z , Song S , Zhang L , Huang Y . 2019. Involvement of jasmonic acid, ethylene and salicylic acid signaling pathways behind the systemic resistance induced by *Trichoderma longibrachiatum* H9 in cucumber. BMC Genomics 20: 144. 30777003 10.1186/s12864-019-5513-8 PMC6379975

[nph71405-bib-0140] Zeier J , Pink B , Mueller MJ , Berger S . 2004. Light conditions influence specific defence responses in incompatible plant–pathogen interactions: uncoupling systemic resistance from salicylic acid and PR‐1 accumulation. Planta 219: 673–683. 15098125 10.1007/s00425-004-1272-z

[nph71405-bib-0141] Zhang C , Ding Z , Wu K , Yang L , Li Y , Yang Z , Shi S , Liu X , Zhao S , Yang Z *et al*. 2016. Suppression of jasmonic acid‐mediated defense by viral‐inducible MicroRNA319 facilitates virus infection in rice. Molecular Plant 9: 1302–1314. 27381440 10.1016/j.molp.2016.06.014

[nph71405-bib-0142] Zhang J , Ren Z , Zhou Y , Ma Z , Ma Y , Hou D , Xu Z , Huang X . 2019. NPR1 and redox rhythm: connections, between circadian clock and plant immunity. International Journal of Molecular Sciences 20: 1211. 30857376 10.3390/ijms20051211 PMC6429127

[nph71405-bib-0143] Zhang X , Jin X , Li J , Dini‐Andreote F , Li H , Khashi U Rahman M , Du M , Wu F , Wei Z , Zhou X *et al*. 2025. Common mycorrhizal networks facilitate plant disease resistance by altering rhizosphere microbiome assembly. Cell Host & Microbe 33: 1765–1778. 40961934 10.1016/j.chom.2025.08.016

[nph71405-bib-0144] Zhao H , Knorr L , Pascucci A , Ma W , Hulin M . 2026. NAD modification at the battlefront in plant‐pathogen interactions. Annual Review of Plant Biology 77: 677–705. 10.1146/annurev-arplant-083123-043500 41770839

[nph71405-bib-0145] Zhou C , Zhu J , Qian N , Guo J , Yan C . 2021. *Bacillus subtilis* SL18r induces tomato resistance against *Botrytis cinerea*, involving activation of long non‐coding RNA, MSTRG18363, to decoy miR1918. Frontiers in Plant Science 11: 4819. 10.3389/fpls.2020.634819 PMC7887324 33613592

[nph71405-bib-0146] Zoeller M , Stingl N , Krischke M , Fekete A , Waller F , Berger S , Mueller MJ . 2012. Lipid profiling of the arabidopsis hypersensitive response reveals specific lipid peroxidation and fragmentation processes: biogenesis of pimelic and azelaic acid. Plant Physiology 160: 365–378. 22822212 10.1104/pp.112.202846 PMC3440211

[nph71405-bib-0147] Zurbriggen MD , Carrillo N , Hajirezaei M‐R . 2010. ROS signaling in the hypersensitive response. Plant Signaling & Behavior 5: 393–396. 20383072 10.4161/psb.5.4.10793 PMC2958590

